# *Pseudomonas* Phage MD8: Genetic Mosaicism and Challenges of Taxonomic Classification of Lambdoid Bacteriophages

**DOI:** 10.3390/ijms221910350

**Published:** 2021-09-26

**Authors:** Peter Evseev, Anna Lukianova, Nina Sykilinda, Anna Gorshkova, Alexander Bondar, Mikhail Shneider, Marsel Kabilov, Valentin Drucker, Konstantin Miroshnikov

**Affiliations:** 1 Shemyakin-Ovchinnikov Institute of Bioorganic Chemistry, Russian Academy of Sciences, 117997 Moscow, Russia; a.al.lukianova@gmail.com (A.L.); sykilinda@mail.ru (N.S.); mm_shn@mail.ru (M.S.); 2 Limnological Institute, Siberian Branch of Russian Academy of Sciences, 664033 Irkutsk, Russia; kovadlo@yandex.ru (A.G.); drucker@lin.irk.ru (V.D.); 3 Institute of Chemical Biology and Fundamental Medicine, Siberian Branch of Russian Academy of Sciences, 630090 Novosibirsk, Russia; alex.bondar@mail.ru (A.B.); kabilov@niboch.nsc.ru (M.K.)

**Keywords:** viral genomics, lambda-like phages, *Pseudomonas* phages, viral taxonomy, bacteriophage evolution, genetic mosaicism, horizontal transfer

## Abstract

*Pseudomonas* phage MD8 is a temperate phage isolated from the freshwater lake Baikal. The organisation of the MD8 genome resembles the genomes of lambdoid bacteriophages. However, MD8 gene and protein sequences have little in common with classified representatives of lambda-like phages. Analysis of phage genomes revealed a group of other *Pseudomonas* phages related to phage MD8 and the genomic layout of MD8-like phages indicated extensive gene exchange involving even the most conservative proteins and leading to a high degree of genomic mosaicism. Multiple horizontal transfers and mosaicism of the genome of MD8, related phages and other λ-like phages raise questions about the principles of taxonomic classification of the representatives of this voluminous phage group. Comparison and analysis of various bioinformatic approaches applied to λ-like phage genomes demonstrated different efficiency and contradictory results in the estimation of genomic similarity and relatedness. However, we were able to make suggestions for the possible origin of the MD8 genome and the basic principles for the taxonomic classification of lambdoid phages. The group comprising 26 MD8-related phages was proposed to classify as two close genera belonging to a big family of λ-like phages.

## 1. Introduction

The genomics of lambdoid (λ-like) bacteriophages has been the focus of scrutiny of several generations of scientists, since the discovery of phage λ by Esther Lederberg, in 1949 [1] and the subsequent explosive growth of interest in this phage [2,3,4,5,6]. The identification of genetic mechanisms responsible for the lysogeny decision, the processes of lysis and adsorption have made a huge contribution to understanding the main principles of the nature of phage infection [7]. Phage λ and related phages remain the focus of virology studies dedicated to the molecular details of the infection cycle of phages [7].

The studies that followed the discovery of phage λ showed the wide range of basic principles that were involved in the genome organisation of this phage among many temperate bacteriophages [8,9,10]. Genomic research has demonstrated the high level of genetic mosaicism of λ-like phages while sharing the same overall gene organisation [11,12,13]. This mosaicism can be caused by homologous recombination that happens when a phage infects a cell carrying a prophage with appropriate homologies [14], or by extensive indiscriminate nonhomologous recombination followed by selection for functional phages [12]. The temperate lifestyle of lambdoid phages and their recombination processes makes them important for bacterial evolution [15] including the adaptation and genomic diversification of bacterial pathogens [16,17].

*Pseudomonas* phage MD8 is a temperate phage with a λ-like genome, which has been obtained from the freshwater of Lake Baikal in 2005 and 2010. Baikal’s viral community is the subject of focused research [18,19]. This phage has been sequenced but has yet to be studied comprehensively. The phage infects *Pseudomonas aeruginosa*, an important opportunistic pathogen that can cause chronic infections leading to significant morbidity and mortality [20,21,22]. Recent studies have discovered new temperate bacteriophages infecting *P. aeruginosa* [23,24]. A GenBank database search has revealed even more unpublished bacteriophages with a lambdoid organisation of the genome. Preliminary comparative bioinformatic analysis of the phage MD8 genome has shown the presence of both genes related to previously described lambdoid phages and genes specific to MD8 or other *Pseudomonas* phages, including possible virulence factors. Analysis has also exposed problems relating to the differentiation and taxonomic classification of *Pseudomonas* phages related to their genetic mosaicism.

Since the first published report in 1971, updating the taxonomic classification of viruses has been the main task of the International Committee on Taxonomy of Viruses (ICTV) [25]. The classification of bacteriophages is a duty of the Bacterial and Archaeal Viruses Subcommittee (BAVS) within ICTV. Since 1971, reports containing the results of thorough studies have been published, but several thousand *Siphoviridae* genomes remain unclassified. This might be due to difficulties with the classification of temperate phages, related to the complex evolutionary history of marker genes and the highly mosaic structure of phage genomes impeding direct genomic alignment [26]. Various clustering approaches have been developed for bacteriophage classification [27], but preliminary studies of MD8 and related phages have shown what appears to be an inconsistency between the results of different techniques.

The current paper introduces the *Pseudomonas* phage MD8 and discusses the difficulties of evolutionary analysis and taxonomic classification of MD8, related phages and λ-like phages in general. First, the biological and morphological properties of MD8 are described. Next, a database search is made for related phages and related genomes are clustered using different methods. Then, essential genes and corresponding proteins are studied using sequence search, and phylogenetic and structural bioinformatic analysis. After that, related genomes are analysed, proteome and protein phylogenies are constructed, the modules acquired by horizontal transfer are found and the possible origins of the mosaic picture of the MD8 genome are reconstructed. Finally, a discussion of the implications of analyses performed and patterns observed for the purposes of taxonomy and classification of lambdoid bacteriophages is presented.

The goal of this research is to find both common and distinctive features of phage MD8 and related λ-like *Pseudomonas* bacteriophages and to offer possible solutions for the taxonomic problems of lambdoids, a group of phages important for virology.

## 2. Results

### 2.1. General Biological Features of Phage MD8

Bacteriophage MD8 was first isolated in 2005, from Lake Baikal freshwater, using *P. aeruginosa* PAO1 as target host strain for the enrichment protocol.

When plated on Petri dishes with two-layer agar (top agar 0.7% *w*/*v*), the bacteriophage formed turbid plaques. After long-term storage in saline, a spontaneous virulent mutant of the temperate phage MD8 was isolated. The virulent mutant produced transparent plaques. To confirm that both types of plaque belong to different forms of the same phage, isolates were analysed according to their structural protein pattern using SDS-PAGE, as well as their DNA restriction patterns by digestion with restriction endonucleases EcoRI and SalI (Supplementary Figure S1). In all cases, the band distribution was identical. All subsequent experiments were performed using a virulent mutant.

The lytic activity of bacteriophage MD8 was tested against 125 uncharacterised *P. aeruginosa* strains typical of Baikal microbial communities and clinical isolates. The phage demonstrated an extremely narrow host range and only infected its isolation host, laboratory strain PAO1.

**Figure 1:**
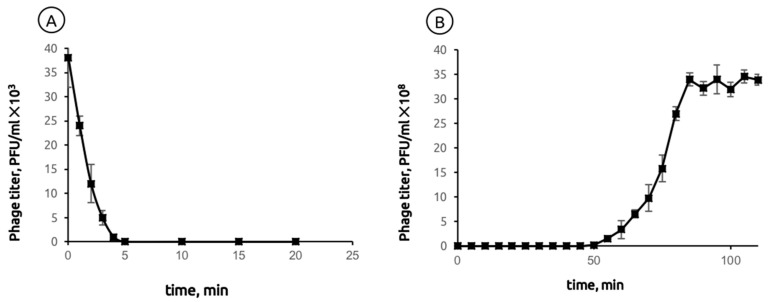
(**A**) Adsorption of phage MD8 to *P. aeruginosa* PAO1. The Y-axis represents the concentration of a non-adsorbed phage in the solution (**B**) Latent period and burst size of phage MD8 on the same host.

The adsorption of phage MD8 to the bacterial host PAO1 at 37 °C occurred relatively quickly. 98% of phage particles bound within four min of incubation at MOI = 0.001 (Figure 1A). A one-step growth experiment showed that the latent period of MD8 was 45 ± 5 min after adsorption and the burst size was 159 ± 10 PFU/cell (Figure 1B).

Phage sensitivity to external conditions, such as temperature, pH, resistance to chloroform and ultraviolet light, was further assayed. Phage MD8 is stable at low temperatures, survives freezing and does not lose activity after prolonged incubation at room temperature and 37 °C. However, heating to 42 °C for 1 h reduced the phage titer by 37% and raising the temperature to 60 °C ultimately inactivated the phage (Supplementary Figure S2A). MD8 was stable in a neutral pH range (pH = 5–7) and a wide salinity range from 0.01 to 5 M NaCl at room temperature (Supplementary Figure S2B,C) and was insensitive to treatment with chloroform for 1 h (Supplementary Figure S2D). A 5-min exposure to UV led to a halving of the titer; 95% of the control particles were inactivated in 10 min (Supplementary Figure S2E).

**Figure 2:**
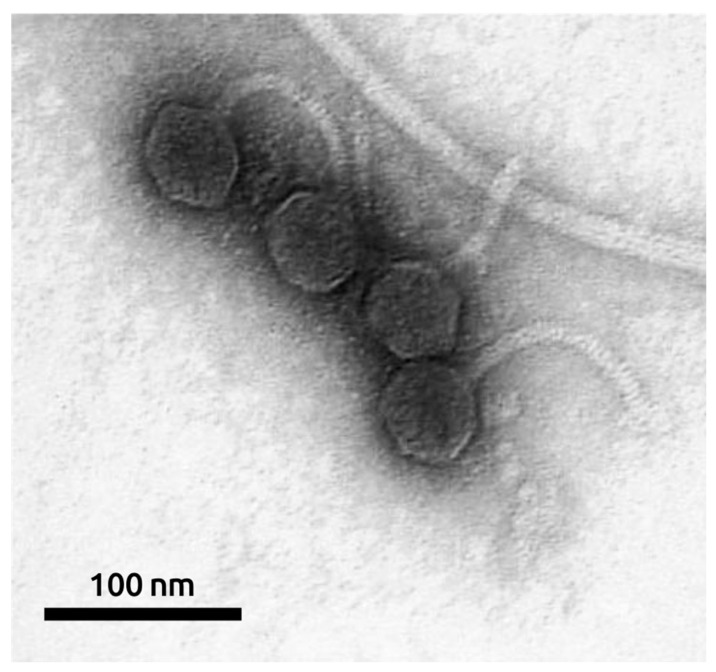
Electron microscopy image of phage MD8. The scale bar is 100 nm.

The morphology of the bacteriophage was studied using transmission electron microscopy. MD8 is a λ-like bacteriophage with a flexible ~120-nm long tail and an isometric capsid with a diameter of ~57 nm (Figure 2).

### 2.2. General Features of Genome and Proteome

*Pseudomonas* phage MD8 (GenBank accession #NC_031091) has a double-stranded genome of 43,277 base pairs. The GC-content of the genome is 61.1% and is distributed evenly through the genome length. The GC-content of *P. aeruginosa* is about 66%, which is slightly higher. The genome of MD8 contains 67 predicted ORFs and contains no tRNA genes (Supplementary Table S1).

**Figure 3:**
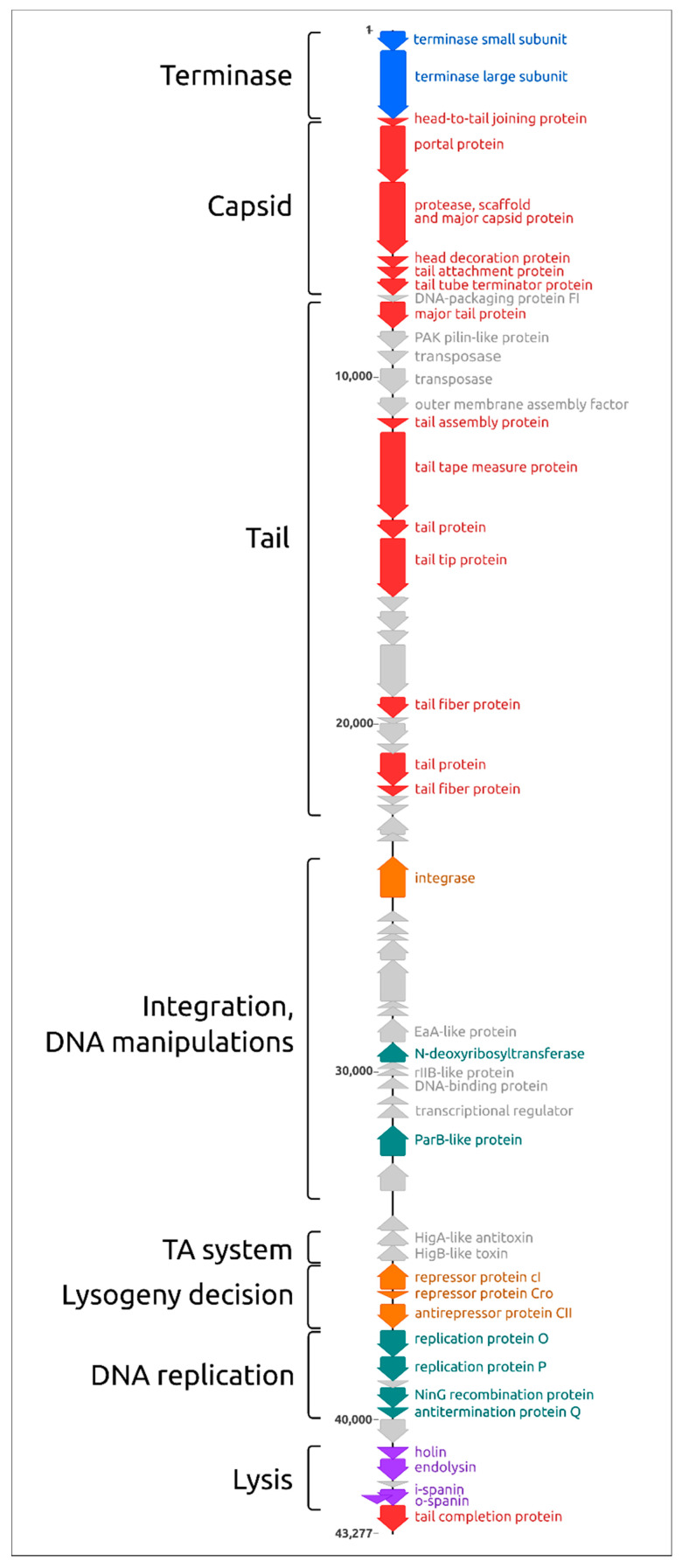
Genetic map of *Pseudomonas* phage MD8 and approximate location of functional modules (left). The colours of genes are as follows: blue, terminase; red, virion; orange, lifestyle; green, DNA manipulations and replication; purple, lysis. The remaining proteins, including the hypothetical ones, are coloured grey.

The structure of the genome (Figure 3) is reminiscent of *Escherichia* phage λ and other lambdoid bacteriophages. Counting from the 5′-end, the genome includes a block of 30 packaging and virion genes oriented in the forward direction, a block of 23 genes, including integrase oriented in the reverse direction and a block of 14 genes, including the replication and lysis genes, oriented in the forward direction again. Interestingly, the major capsid protein is encoded together with protease by a single gene 5.

A sequence search of MD8 predicted proteins using the GenBank Bacterial database (BLASTP, E-value < 10^−5^) testified to the presence of homologous sequences in various bacterial genomes. Homologs of all 67 proteins were found in bacterial sequences. Homologs of 13 proteins were found in *Pseudomonas* species only. Homologs of 54 proteins including lysogeny proteins were also found both in genomes of *Pseudomonas* bacterial strains and in other bacteria. These findings point to a long evolutionary history for *Pseudomonas* phage MD8 and its association with the temperate lifestyle.

To ascertain the closest relatives of phage MD8, the BLAST database made up of all 14,983 phage genomes contained in GenBank up to May 2021 was used, and a sequence search with predicted MD8 proteins was conducted. The search indicated that 22 *Pseudomonas* Siphoviruses, including phage MD8, often group together distantly from other phages, share significant homology in a majority of MD8 proteins and possess a similar GC-content of 61.1–62.5%. These phages are: MD8, F10, JBD68, PAN70 [28] and vB_Pae_BR133a, vB_Pae_BR144a, vB_Pae_BR200a, vB_Pae_BR204a, vB_Pae_BR213a, vB_Pae_BR233a, vB_Pae_BR299a, vB_Pae_CF24b, vB_Pae_CF28b, vB_Pae_CF34a, vB_Pae_CF55b, vB_Pae_CF60a, vB_Pae_CF67a, vB_Pae_CF79a, vB_Pae_CF140a, vB_Pae_CF145a, vB_Pae_CF165a and vB_Pae_CF208a found by metagenomic methods from chronic *Pseudomonas aeruginosa* lung infections [29]. Furthermore, proteins of *Pseudomonas* phages φ2 [30] and TC7 share a high level of similarity with MD8 proteins, except for the group of gene products 1–9 and several other proteins. Several MD8 proteins are clustered together with other *Pseudomonas* phages. Only one MD8 hypothetical protein (gene product, gp32) was not found encoded in the genomes of other phages.

### 2.3. ANI and VIRIDIC Clustering

Calculations of average nucleotide identity (ANI) of all 14,983 phages deposited in the GenBank phage database up to May 2021, using orthoANIu, revealed 25 phages clustering together with MD8 and demonstrating the aligned fraction of more than 10%, which is significantly higher than for all other phages (Table 1, Supplementary Table S2). The ANI numbers for those were about 89–96%, compared to phage MD8. This range is higher than the nucleotide identity genus threshold of 70% and close to the species threshold of 95%. However, the low aligned fraction of the genome does not seem to allow for reliable taxonomic conclusions.

**Figure 4:**
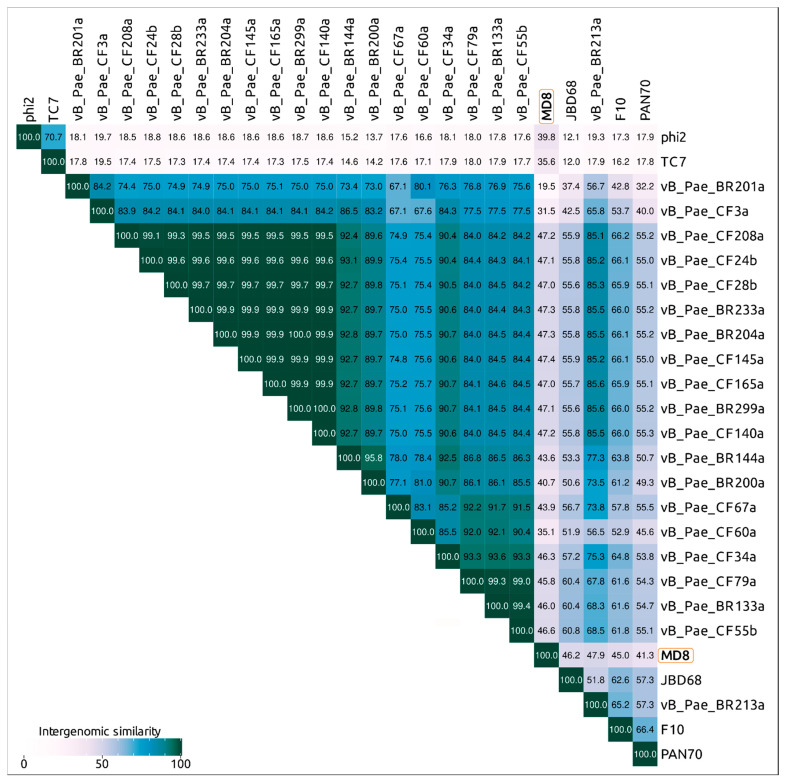
VIRIDIC generated heatmap of 26 “MD8-like” *Pseudomonas* phages. The colour coding indicates the clustering of the phage genomes based on intergenomic similarity. The numbers represent the similarity values for each genome pair, rounded to the first decimal.

The Virus Intergenomic Distance Calculator (VIRIDIC) server offers an implementation of the traditional algorithm used by the ICTV and possesses a number of advantages [31]. An analysis of VIRIDIC data obtained with different sets of phages, including all *Pseudomonas* Siphoviruses, also highlighted a group of 25 *Pseudomonas* phages comparatively closely related to phage MD8 (Figure 4, Supplementary Figures S3 and S4). The list of these phages includes phages CF3a and BR201a. The latter two phages are represented by partial genomes lacking the terminase, major capsid protein and some other conservative genes, but their proteins share homology with several other MD8 proteins.

According to the VIRIDIC and ANI calculations, the phages vB_Pae_BR204a, vB_Pae_BR233a, vB_Pae_BR299a, vB_Pae_CF24b, vB_Pae_CF28b, vB_Pae_CF140a, vB_Pae_CF145a, vB_Pae_CF165a and vB_Pae_CF208a have an intergenomic similarity of 99% and higher and can be considered as races of the same species. Bacteriophages vB_Pae_BR133a, vB_Pae_CF55b and vB_Pae_CF79a also represent the races of the same species with intergenomic similarity of 99% and higher.

In the following discussion, a group of 26 phages listed in Table 1 and Figure 4 will be referred to as “MD8-like phages” or “MD8 group”.

### 2.4. Terminase Phylogeny

**Figure 5:**
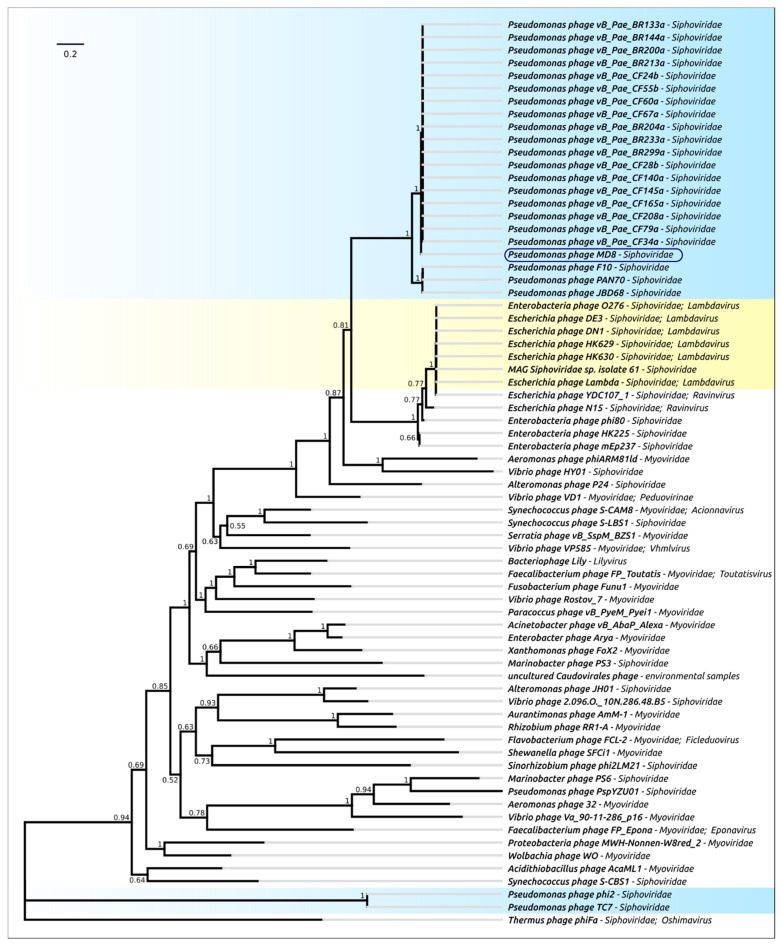
Phylogenetic tree obtained with MRBAYES, based on terminase large subunit amino acid sequences. Bayesian posterior probabilities are indicated near their branches. The taxonomic classification is shown to the right of the phage’s name. The scale bar shows 0.2 estimated substitutions per site and the tree was rooted to *Thermus* phage ϕFA1. The chain length was 7,700,000, the burn-in length was 700,000, the subsampling frequency was 200 and the average standard deviation of split frequencies was 0.0069.

Terminase is one of the most conservative bacteriophage proteins and is often used for the purposes of taxonomic attribution [32]. To construct the terminase phylogenetic tree, the predicted amino acid sequence of terminase large subunit was searched with BLASTP (E-value < 10^−3^), using a custom phage protein database built with all proteins predicted in all 14,983 GenBank genomes. To construct the resulting phylogenetic tree with 75 representatives of bacteriophages (Figure 5), the intermediate trees were analysed and phages representing all the adjacent and main clades were selected. The terminase sequence of *Thermus* phage ϕFA1 was added as an outgroup.

The tree indicates the close relatedness and common ancestry of the terminases of 22 phages of the MD8 group and the λ-like *Escherichia* phages, including the phages of *Lambdavirus* and *Ravinvirus* genera. Interestingly, the P2-like *Vibrio* phage VD1 Myovirus terminase is not very distant from the MD8 and lambdoid terminase. In this tree, Myoviruses and Siphoviruses are not monophyletic groups. The tree may also reflect the complex multiple origin of bacteriophages with different morphology and the horizontal transfer of terminase genes. Surprisingly, the terminases of phages φ 2 and TC7 are positioned closer to the *Thermus* phage phiFA1 terminase than to the terminase of *Lambdavirus* phages and MD8.

In this way, the terminase phylogeny suggests the relatedness and monophyleticity of 22 MD-8 phage terminases and their closeness to the terminase of phages λ and N15. The tree also indicates the distant position of the φ1 and TC7 terminases from the remaining MD8-like phages.

### 2.5. Major Capsid Protein: Gene, Structure and Phylogeny

For all *Pseudomonas* phages sharing the MD8 terminase phylogenetic tree clade (Figure 5), where the coding sequences of the major capsid protein (MCP) were predicted, the gene product (MD8 gp5 for MCP) seemingly possess a multi-domainal structure. As was confirmed by BLASTP, PSI-BLAST and HMM searches, the N-terminal domain of MD8 gp5 has a strong similarity with viral capsid maturation protease, and the C-part is structurally similar to HK97-fold containing major capsid proteins. This feature differentiates most MD8-like phages from phages φ1 and TC7.

In *Duplodnaviria Heunggongvirae* viruses encompassing tailed bacteriophages and herpesviruses, the capsid is built by assembling the major capsid protein with a scaffolding protein with the formation of intermediate procapsids. After the construction is complete the scaffold proteins escape from the capsid [33,34,35]. Many phages, including *Escherichia* phage HK97 [36], bacteriophage λ [37] and phages Mu [35], P2 [38] and T4 [39] employ a capsid maturation protease that degrades the parts of the procapsid to assist the scaffolding protein to leave the capsid. In some cases (e.g., phages T7 [40], P22 [41], and φ 29 [42]), the phages do not need the aid of a protease to enable their scaffolding proteins to be released. Some other phages, including HK97, do not have separate scaffolding proteins. In phage HK97, the N-terminal 102 amino acid residues form a so-called ‘delta domain’, which performs the role of the scaffolding protein and is further degraded by the protease [36]. Gene 5 encoding the HK97 MCP is located immediately after gene 4 encoding the capsid maturation protease. In temperate phages like P2 [38] or λ [43], the protease and scaffolding protein are encoded by a single gene.

**Figure 6:**
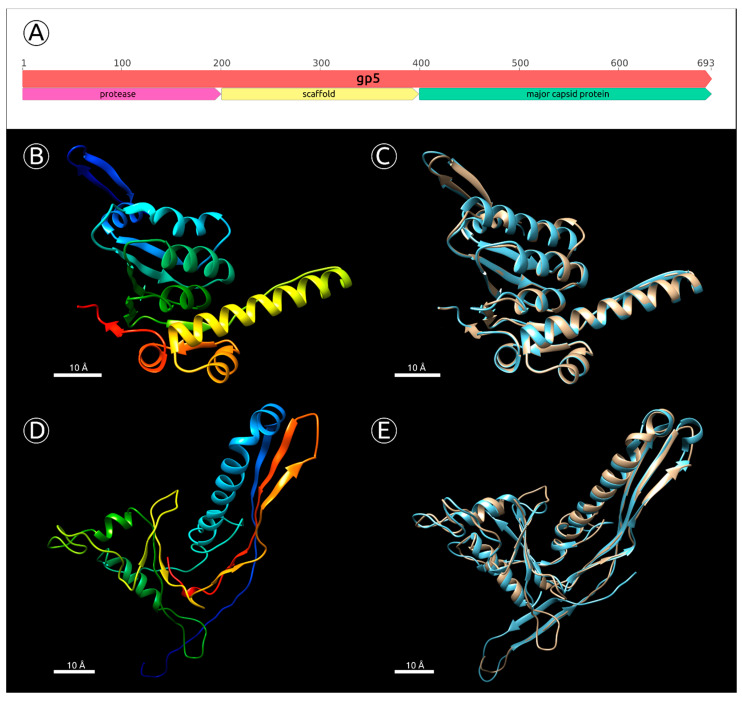
(**A**) Approximate positions of the gene 5 domains encoding protease, scaffolding protein and major capsid protein. (**B**) Predicted by the homology modelling tertiary structure of MD8 protease domain (gp5 amino acid residues 16–204, confidence 100%, template PDB accession 3Q7H). (**C**) Structural alignment of the predicted MD8 protease domain and ClpP subunit of the ATP-dependent Clp Protease from *Coxiella burnetii* used as a template. (**D**) Predicted by the homology modelling tertiary structure of MD8 MCP domain (gp5 amino acid residues 456–690, confidence 100%, template PDB accession 1IF0). (**E**) Structural alignment of the predicted MD8 protease domain and Bacteriophage HK97 procapsid used as a template. The scale bar is 10 Å.

It seems that, in the case of MD8-like phages, there is a situation whereby all three proteins—capsid maturation protease, scaffold protein and MCP—are encoded by a single gene. This suggestion is supported by the following:

- BLAST and HMM-motif searches found significant similarities between the N-terminal part of MD8 gp5 and phage capsid maturation bacterial and other proteases. The length of MD8 gp5 was 693 residues. The Phyre2 modelling server estimated the range of the proteolytic region aligned with PDB structures of known proteases to be about 15–210 amino acid residues of gp5, with 100% confidence. The HHpred HMM search aligned the MD8 gp5 with the protease region of the protease and scaffolding protein of phage λ, with 99.9% probability (Supplementary Figure S5);
- The BLAST and HMM comparison demonstrated significant similarities between the C-terminal part of MD8 gp5 and major capsid proteins of various bacteriophages. Phyre2 modelled the residues 456–690 of MD8 gp5 with bacteriophage HK97 procapsid II, with 98.5% confidence (Figure 6D,E), and the HHpred search had a 98.6% probability. HHpred also pointed to the major capsid protein of *Escherichia* phage Mu being the closest relative, with a probability of 99.9% and an E-value of 1.3 × 10^−28^;
- Analysis of the HMM alignment and modelling of the secondary structure demonstrated that the C-terminal part of the phage λ protease and scaffolding protein, containing the scaffolding domain, possessed a similar secondary structure with MD8 gp5 for 80% of the λ scaffolding domain, except for 40 final C-terminal residues. Furthermore, no homologs or similar scaffolding proteins were found by the search of all other predicted proteins of phage MD8.

Having performed a more thorough comparative analysis of the data obtained, and with the suggestion that the scaffolding domain of the MD gp5 had the same length as that of phage λ gp5, it can be assumed that the approximate locations of domains were as shown in Figure 6A. It is possible to suggest that the structural similarities found with phages λ and Mu (where protease and scaffold were encoded by a single gene and MCP was encoded by a separate gene) may indicate that the MD8 gene 5 originated from the fission of the protease-scaffold gene and MCP gene.

**Figure 7:**
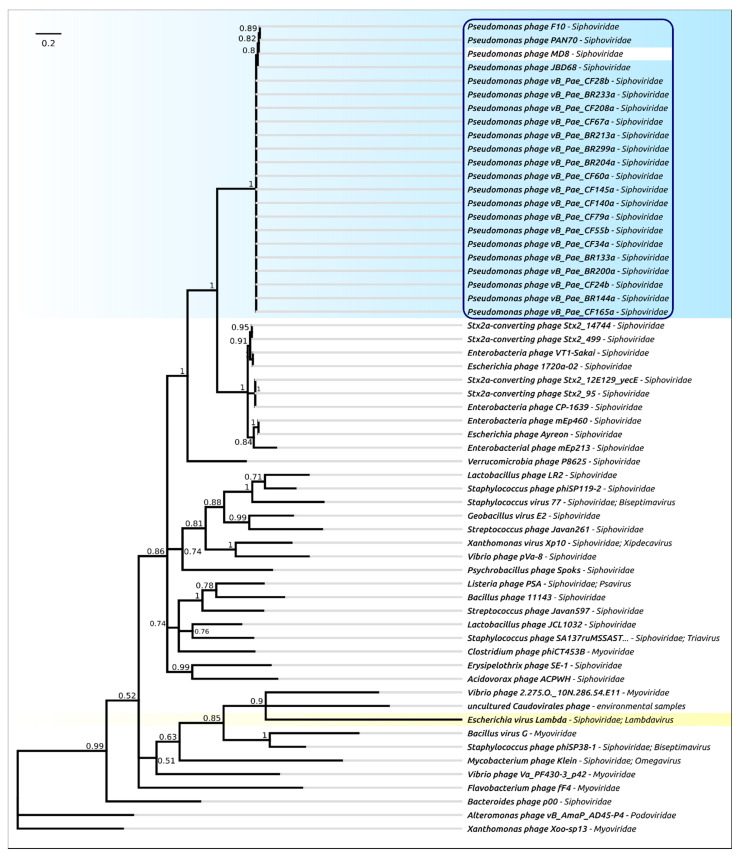
Phylogenetic tree obtained with MRBAYES, based on protease domain amino acid sequences. Bayesian posterior probabilities are indicated near their branches. The taxonomic classification is shown to the right of the phage’s name. The scale bar shows 0.2 estimated substitutions per site and the tree was rooted to phage *Xanthomonas* phage Xoo-sp13. The chain length was 2,200,000, the burn-in length was 200,000, the subsampling frequency was 200 and the average standard deviation of split frequencies was 0.011.

**Figure 8:**
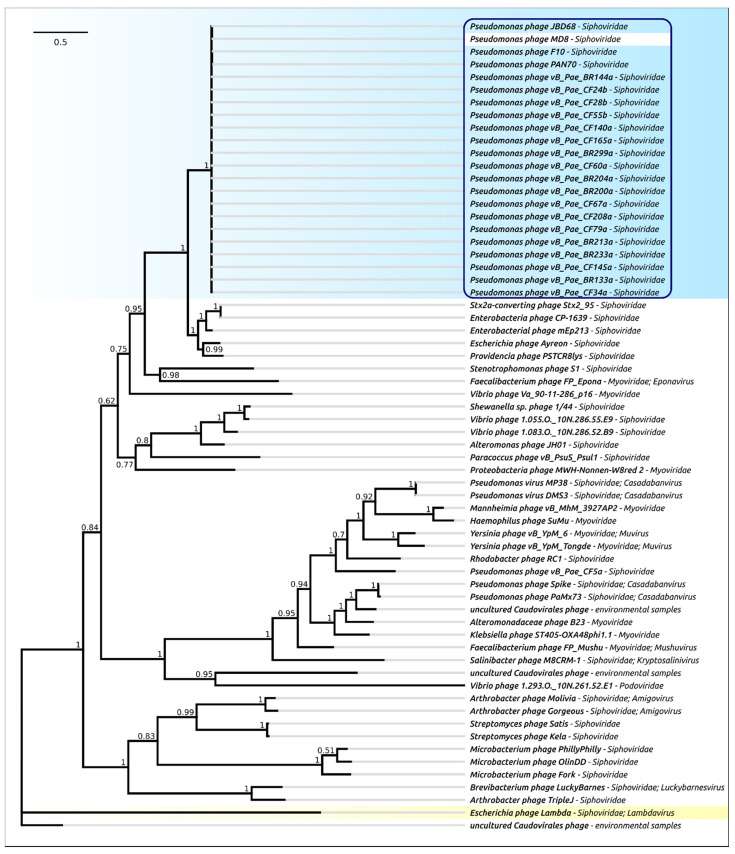
Phylogenetic tree obtained with MRBAYES, based on the major capsid protein domain amino acid sequences. Bayesian posterior probabilities are indicated near their branches. Taxonomic classification is shown to the right of the phage’s name. The scale bar shows 0.2 estimated substitutions per site and the tree was rooted to phage λ. The chain length was 2,200,000, the burn-in length was 200,000, the subsampling frequency was 200 and the average standard deviation of split frequencies was 0.007.

The phylogenetic analysis was performed in a similar way to that for the terminase, but separately for the protease and MCP domains (Figure 7 and Figure 8). As in the case of terminase, the protease and MCP phylogeny shows a different evolutionary history for φ2 and TC7 on the one hand, and MD8 and other members of the MD8 group on the other hand. It is noticeable that the phylogenetic analysis of the protease and MCP domains does not show close evolutionary relationships between the proteins of MD8-like phages (including TC7 and φ2) and phage λ. However, both trees indicate a similar recent evolutionary history for both domains in all MD8-like phages analysed. These findings might indicate that the recombinational exchange with the terminase genes does not necessarily require the participation of the adjacent protease and MCP genes, while such an exchange with the MCP genes or domains may proceed only with the protease gene (or domain) simultaneously. It might also be hypothesised that the events of fission of the protease, scaffold and MCP genes can bring evolutionary advantages, resulting in the more productive emergence of functional phages.

### 2.6. Replication Proteins

The list of predicted replication proteins of phage MD8 includes proteins that are distant from proteins of phage λ by their primary structure, but similar to them by their secondary structure, as revealed by an HMM motif comparison. In bacteriophage λ, only two proteins, O and P, participate in the initiation and propagation of the replication forks [44]. Four dimers of O protein bind at four sequence repeats in the origin oriλ, the λ origin of replication located midway within the O sequence, to form an “O-some” complex [45]. The replication protein P interacts with the host DnaB helicase, and this complex binds the O-some to form the ori:O:P:DnaB preprimosomal complex [46]. Some lambdoid phages encode a putative DNA polymerase [47] or a bifunctional DNA primase/helicase [48]. Like phage λ, phage MD8 has no DNA polymerase genes and relies on the host DNA replication machinery [9]. The cluster of MD8 replication proteins includes both O-like (gp56) and P-like (gp57) proteins. The predicted similarity of replication proteins and the presence of repeats within the gene encoding the O-like protein, as well as the absence of DNA polymerase and helicase-primase genes, confirms the correctness of the predictions made. It seems that the replication mechanism of phage MD8 is basically similar to that of phage λ.

The MD8 genome also encodes a homolog of the NinG protein of phage λ (MD8 gp59). The λ NinG participates in RecBCD recombination pathways of *E. coli* operating on phage λ [49,50]. Interestingly, the replication protein sequences of phage MD8 demonstrate a high level of similarity with φ2 and TC7 replication proteins but do not share any noticeable homology with proteins of all remaining members of the MD8 group. Besides, 19 MD8-like phages that do not demonstrate a similarity of replication proteins with those in the MD8 group possess +DNA primase/helicase genes. This indicates horizontal transfer events in the evolutionary history of a portion of MD8-like phages associated with the loss of the entire module of replication proteins.

The phylogenetic trees of the predicted replication proteins are shown in Supplementary Figures S6–S8.

### 2.7. Lysogeny and Integration Proteins

As was found by the BLASTP search, all members of the MD8 group, including phage MD8, encode relatively close homologs of the phage λ CI, which is well known for being responsible for the maintenance of lysogeny (MD8 gp53) [51]. The genomes of MD8 and a portion of related phages also contain similar genes of CI competitor Cro (MD8 gp54) [52]. It was thought that CI protein performs an important function in the maintenance of the lysogenic state of lambdoid phages [51,53], but further detailed studies have shown that another transcriptional activator, CII, is primarily responsible for the establishment of lysogeny [7]. Homologs of CII were found in all phages of the MD8 group (MD8 gp55). In the present study, it was not possible to identify a homolog or an analog of protein CIII in the genomes of MD8-like phages. This protein is responsible for stabilisation of CII [54] and inhibits protease FtsH from participating in the control of the λ lysis-lysogeny decision by rapid degradation of CII [55]. It is likely that the establishment of lysogeny in MD8 has its distinctive peculiarities or an analog of the λ CIII is very distinct from the proteins described.

The presence of a gene encoding a plasmid-like ParB-like protein (MD8 gp48) was also detected. This protein plays an important role in the reproduction of temperate bacteriophages existing as plasmids in the host cell [56]. Some λ-like phages exist as linear plasmid prophages [7]. However, The BLAST search using the GenBank phage database failed to find homologs of the MD8′s ParB-like proteins in the genomes of temperate λ-like or other phages that were published and described as existing as plasmids in the host cell. It seems that details of MD8 lifestyle and replication need clarification.

The phylogenetic analysis placed CI, CII and Cro proteins of phages MD8 and φ2 in a distinct clade relatively distant from all the remaining MD8-like phages where homologous sequences have been found (Supplementary Figures S9–S11). It is noteworthy that the topology of the trees differs from the topology of the phylogenetic trees of adjacent genes. Meanwhile, the amino acid sequence of the integrase of phage MD8, which is located several genes downstream of the genes for maintaining lysogeny, demonstrated a level of homology similar to phages φ2 and TC7, and the topology of the phylogenetic tree placed TC7, φ2 and MD8 in a clade separate from other MD8-like phages (Supplementary Figure S12). This indicates recombinations resulting in the replacement of the entire module containing the lysogeny maintenance genes.

### 2.8. Adsorption Apparatus

The adsorption apparatus of tailed bacteriophages comprises receptor-binding proteins (RBPs), regularly including tail fibres (tail spikes) as primary RBPs [57]. Other structural proteins, including baseplate proteins [58] and decoration proteins [59] can facilitate the adsorption. In phage λ, the gene product J makes up the distal tip structure of the tail and constitutes the phage RBP [60]. The λ protein J interacts with the bacterial outer membrane maltoporin LamB [61]. The λ wild type strain commonly used in a laboratory has no tail fibres, because of a frameshift mutation in *stf* gene relative to Ur-λ, the original isolate, but the original phage possesses thin tail fibres, which are important for adsorption to the host cell expanding the host range and accelerating the adsorption [62].

The phage MD8 genome does not encode proteins homologous to the λ tail proteins. However, the HMM-HMM comparison has revealed similarities of putative tail proteins with the tail proteins of other phages, suggesting functions for several ORFs of MD8 as tail proteins. The function of MD8 gp18 seems to be analogous to λ protein J tail tip protein, and two proteins encoded by genes 23 and 28 appear to constitute the phage tail fibres. Sequence comparison and phylogenetic analysis (Supplementary Figures S13–S15) indicated the presence of tail tip and tail fibre homologs in *Pseudomonas* phages belonging to different groups, not just the MD8 group. Interestingly, while the tail tip protein homologs were found only in lambdoid phages, the presence of tail fibre homologs was detected in distant groups including Podoviruses.

### 2.9. Lysis Machinery

The lysis apparatus of phage MD8 is generally similar to λ in terms of the functionality of proteins involved but differs in the origin of the proteins. As well as the lysis apparatus of phage λ, the MD8 lysis cassette includes holin (gp62), endolysin (gp63) and inner and outer spanins (gp65 and gp66) [63,64]. There is also a gene encoding a hypothetical protein located between the endolysin and inner spanin genes, but it was not possible to assign a function for that gene. The amino acid primary sequence of holin demonstrated a distant similarity with the λ holin (with a pairwise identity of about 31%), whereas the other three proteins did not show any significant homology with proteins of the *Lambdavirus* phages. Unlike λ spanins, which were encoded by the adjacent genes, the MD8 outer spanin gene was nested in the inner spanin gene, as with some other phages [65,66].

The phylogenetic trees (Supplementary Figures S16–S19) show a close relatedness of lysis proteins belonging to various *Pseudomonas* phages. The trees also expose a less than identical topology for different lysis proteins. It may be suggested that, unlike the genes encoding capsid proteins, the lysis genes are more prone to recombinations alone.

### 2.10. Toxin-Antitoxin System, Virulence Factors

Two genes encoding the toxin (MD8 gp52) and antitoxin (MD8 gp51) were predicted using the HMM motif comparison. The MD8 toxin-antitoxin (TA) system appears to be similar to the Type II HigB-HigA system found in many pathogens, including *P. aeruginosa* [67], and shares an unusual and intrinsic for HigB-HigA TA gene arrangement where the toxin gene *higB* is located upstream of the antitoxin gene *higA* [68]. HigB toxin belongs to the RelE toxin superfamily. It cleaves mRNAs preferentially at stop codons in the ribosomal A site while the antitoxin HigA reduces the activity of toxin HigB by binding to the TA operon promoter. Close homologous sequences were found in several *P. aeruginosa* strains and *Pseudomonas* φ2 Siphovirus.

The genome of phage MD8 encodes pilin (gp11) structurally similar to *P. aeruginosa* PAK pilin (Phyre2 confidence 99.6%, coverage 79%) [69]. It can play an important role in phage infection. Thousands of pilin copies assemble in type IV pili, which are required for the virulence of some Gram-negative bacterial pathogens. Bioinformatic analysis indicated the presence of the transmembrane region in gp11. The gene11 is neighboured by transposases related also to a number of temperate phages, including *Shigella* SfX Siphovirus, *Ralstonia* RsoM1USA P2-like Myovirus, Stx2-converting phages, that can indicate a lateral transfer of pilin with the participation of those phages and a bacterial host. Homologs of pilin were found only in phages JBD68 and PAN70, belonging to the MD8 group, and in *Pseudomonas* chromosomes and plasmids. The homologs of antitoxin were found in φ2 and *Pseudomonas* phages MR13/MR15, not belonging to the MD8 group.

### 2.11. Proteome Analysis and Comparisons with Related Phages

**Figure 9:**
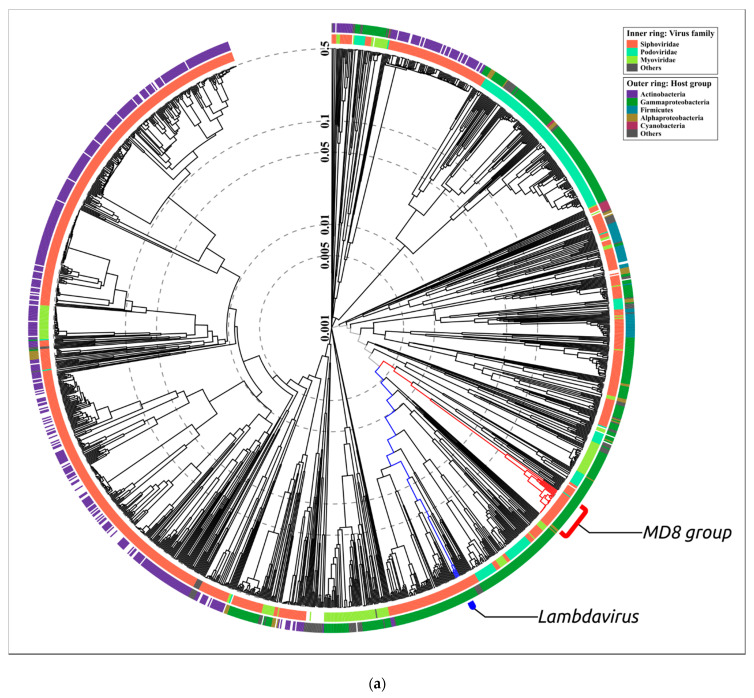

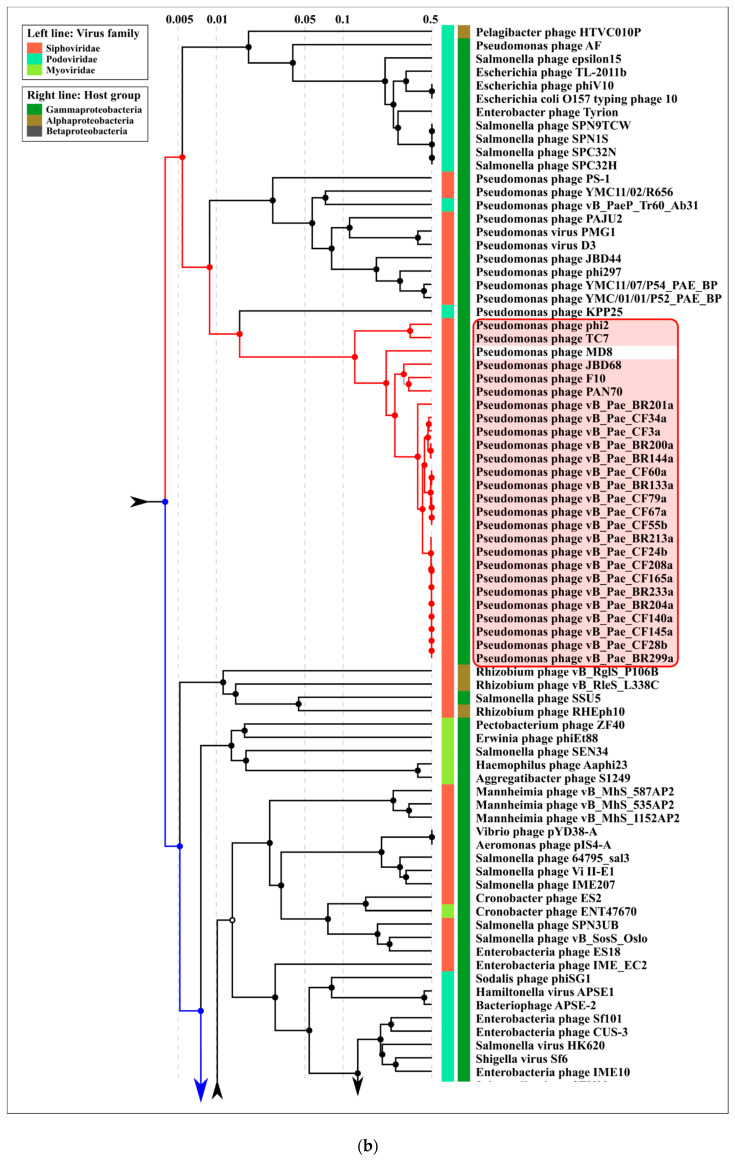

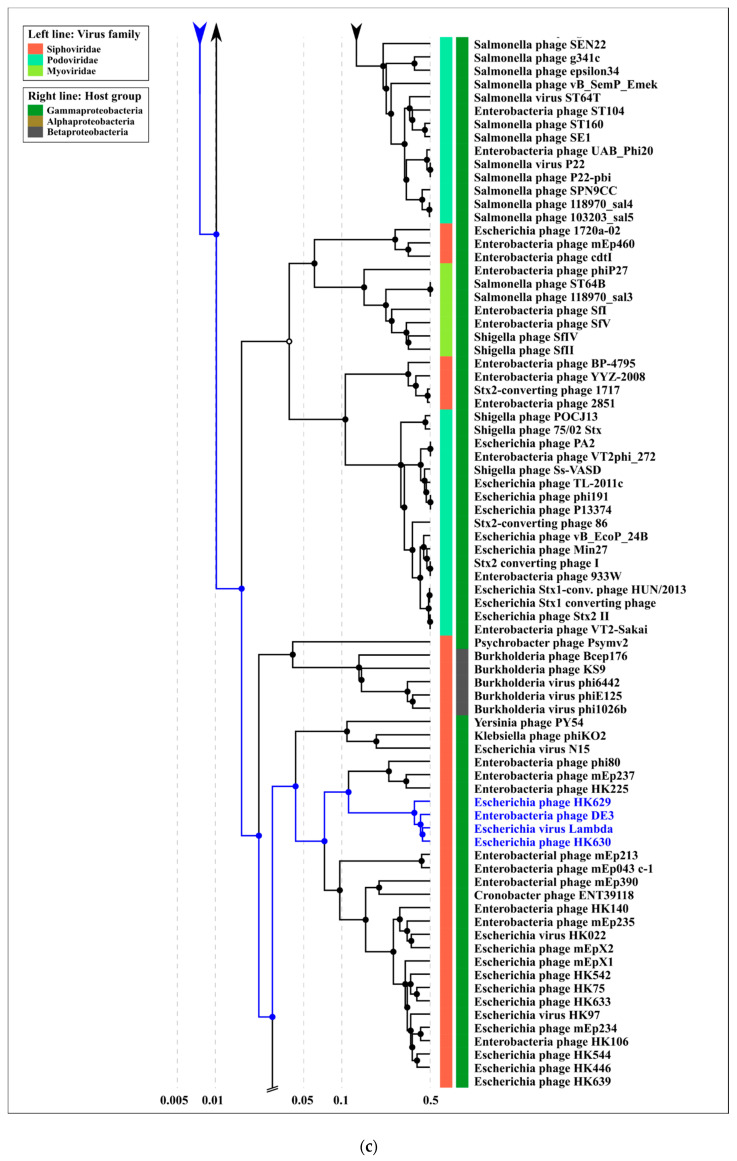

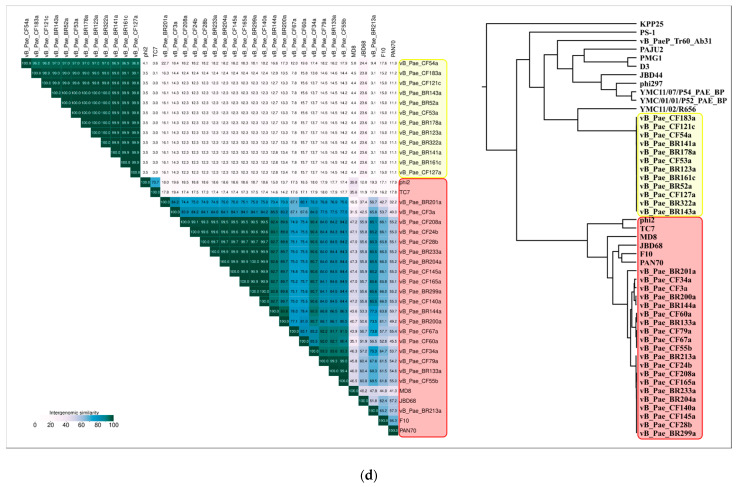
(**a**) Circular proteomic dendrogram plotted by ViPTree using phage genomes of 26 MD8-like phages and genomes found by ViPTree to be related to MD8-like genomes. (**b**,**c**) The clade of the proteomic tree containing MD8-like phages and phage λ. (**d**) The VIRIDIC matrix (left) and ViP dendrogram using an extended set of *Pseudomonas* phages.

The proteome analysis was also conducted using the ViP server (https://www.genome.jp/viptree/ (accessed on 14 June 2021)), exploring phage genomes and estimating predicted genes’ similarities as computed by TBLASTX. The approach used by ViP server algorithms may be more suitable for taxonomic purposes in situations when conserved genes among analysed genomes are absent and intensive genome rearrangements, including horizontal gene transfer, are observed [70]. The proteomic trees constructed using the ViP server are shown in Figure 9. The dendrograms indicate the overall similarity of 26 MD8-like proteomes, placing these phages in one clade. They also demonstrate a slight similarity between proteomes of MD8 group and *Lambdavirus* phages. The tree suggests that phages TC7 and φ2 are related phages which have diverged before the other members of MD8 group. The clustering made by ViPtree seems to be consistent with the VIRIDIC clustering (Figure 9D).

According to the proteomic tree, the *Lambdavirus* group diverged later and the formation of the lambdoid phage groups was accompanied by genetic exchange with very different phages, including Myoviruses. However, the contradictory history of the formation of the lambdoid genomes does not necessarily lead to certain conclusions about the evolutionary history of the genomes. In addition, the consistency of ViPtree is questionable, because the topology of the tree depends on a set of phages (comparing the topology of Figure 9B,D trees).

**Figure 10:**
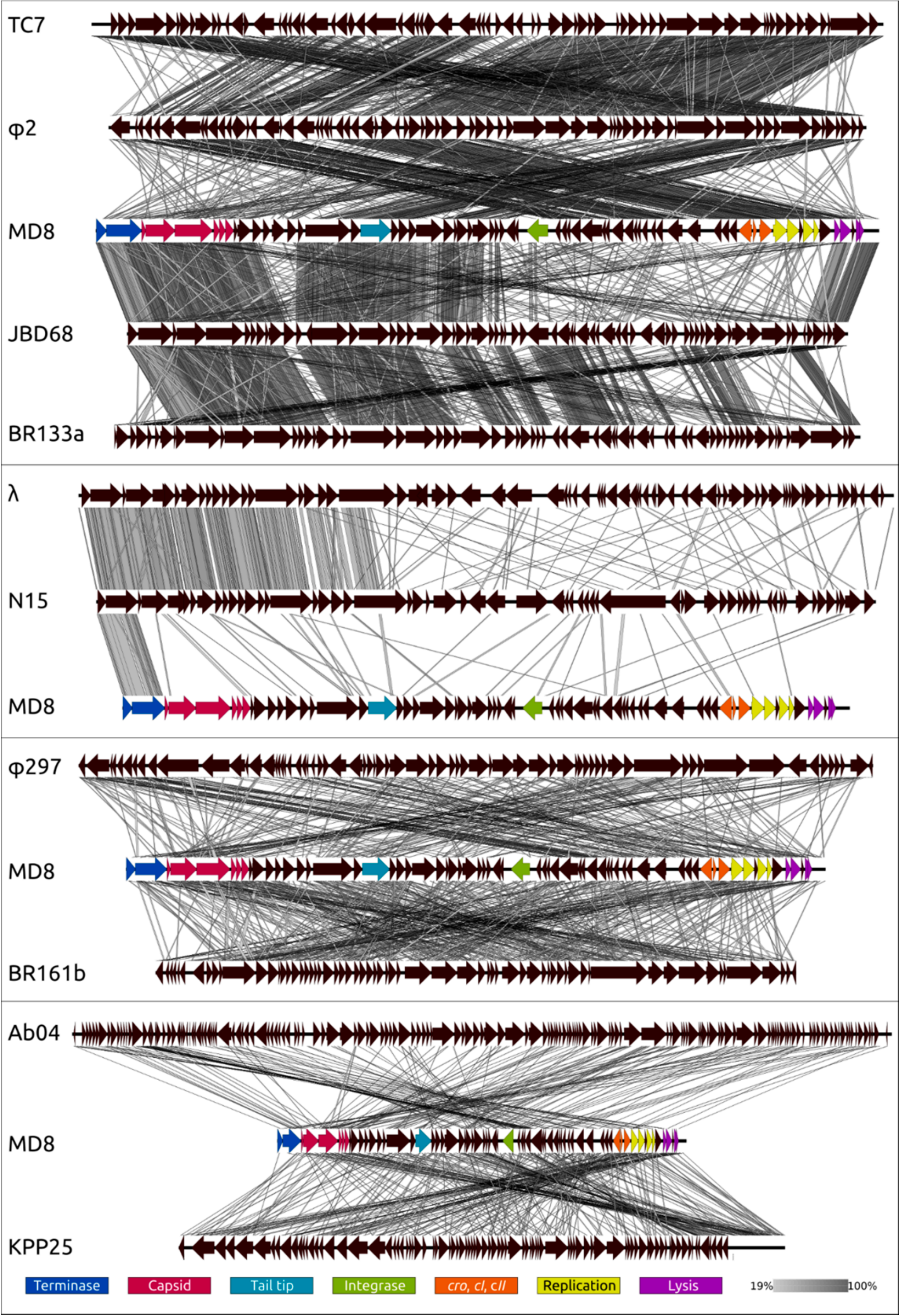
Genome sequence comparison among phage genomes exhibiting co-linearity detected by TBLASTX. Phage abbreviations are as follows: TC7—*Pseudomonas* phage TC7, φ2—*Pseudomonas* phage φ2, MD8—*Pseudomonas* phage MD8, JBD68—*Pseudomonas* phage JBD68, BR133a—*Pseudomonas* phage vB_Pae_BR133a, λ—*Escherichia* phage λ, N15—*Escherichia* phage N15, φ297—*Pseudomonas* phage φ297, BR161b—*Pseudomonas* phage vB_Pae_BR161b, Ab04—*Pseudomonas* phage vB_PaeM_PAO1_Ab04, KPP25—*Pseudomonas* phage KPP25. The percentage of sequence similarity is indicated by the intensity of the grey. Vertical boxes between the analysed sequences indicate areas with a similarity of at least 19%, shown on the scale below. The MD8 genes are coloured according to their function in the legend.

A comparison was also made of the genomes of different phages to reveal the similarities between whole genomes and parts of genomes (Figure 10, Supplementary Figure S20).

The genome comparison of MD8 with MD8-like phages (Figure 10, upper scheme) demonstrates that the formation of the MD8 genome is related to a loss of 5′-end genes presented in TC7 and φ2 genomes, and a similar situation is observed with gene blocks of MD8-like phages JBD68 and vB_Pae_BR133a. It appears that this is an exchange for genes of the same functionality but which have a distinct origin. A similar situation is detected for phages λ and N15 (Figure 10), but the recombination has occurred at another site. Analogous recombination involving a Podoviral phage was reported earlier, with another *Pseudomonas* λ -like phage [71]. This demonstrates that the new lambdoid bacteriophages can regularly arise as a result of the fission of two phage parts. The genomic comparisons of MD8 with lambdoid phage vB_Pae_BR161b, not belonging to MD8 group, non lambdoid Siphovirus φ297, Myovirus vB_PaeM_PAO1_Ab04 and Podovirus KPP25 indicates that genetic exchanges can involve even distantly related phages.

### 2.12. Phylogeny of Proteins and Mosaicism of MD8 Genome

The structure and possible origin of the MD8 genome was studied using phylogenetic analysis and sequence comparisons of all MD8 proteins. The predicted protein sequences of phage MD8 were used for the BLASTP search with the phage GenBank database (E-value < 10^−3^). If the number of homologs found was three or fewer, the pairwise identity values were used for the elucidation of the most related homologous sequence, otherwise, the phylogenetic trees were used for finding the related sequences. In addition, the MD8 proteins were tested for the presence of homologs in bacterial genomes (BLASTP with E-value < 10^−5^ using the GenBank bacterial database).

**Figure 11:**
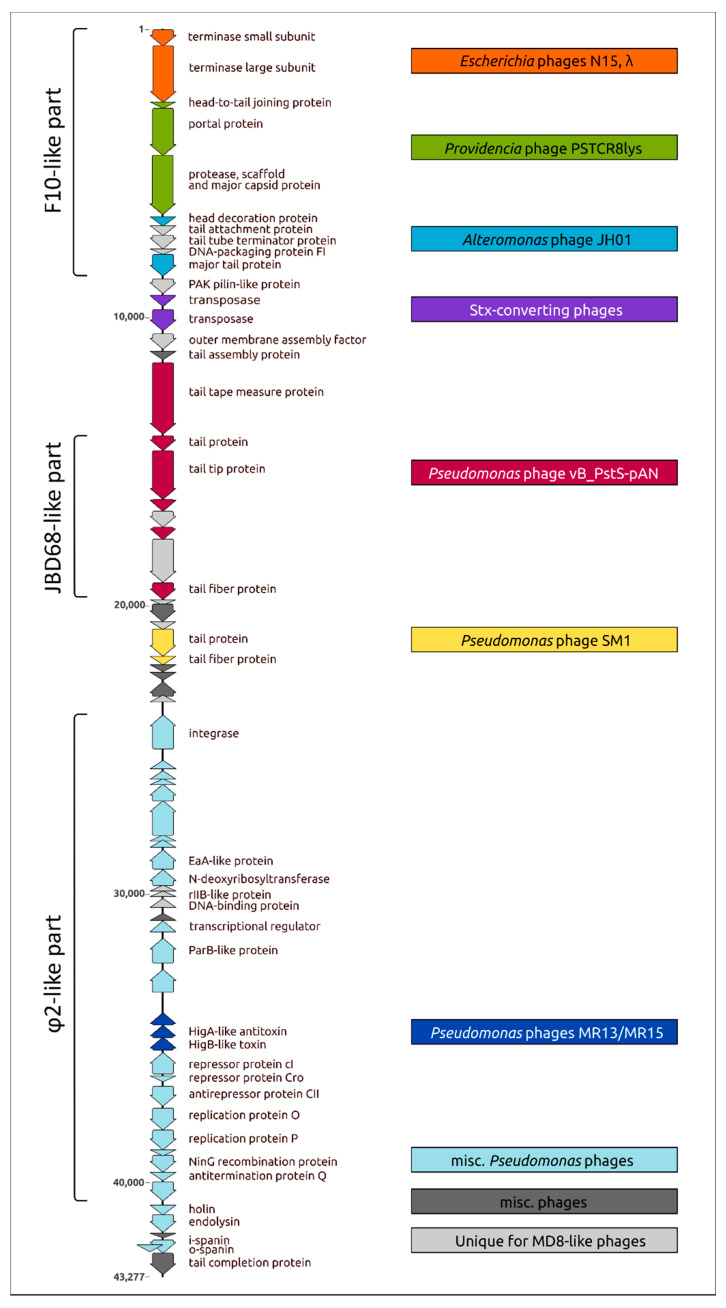
Genetic map of the Pseudomonas MD8 phage and genomic modules identified by sequence comparison and phylogenetic analysis. Parts of the genomes generally more similar to the F10, JBD68 and φ phages, which belong to the MD8 group, are shown in parentheses on the left. The MD8 genes are coloured corresponding the colours of boxes on the left, which contain the names of not MD8-like phages related to MD8 according to analyses of proteins encoded by these genes.

The phylogenetic analysis pointed to the complex and multi-stage nature of MD8 genome shaping. The results of the analysis are consistent with the results of the genomic comparison and indicate that the 5′-part of the MD8 genome including the terminase and capsid genes 1–10 has an origin that is distinct from most other genes. Most of the proteins of this block are more similar to a group of phages including MD8-like phage F10 than to phages φ2 and TC7. The latter two phages contain similar terminase and capsid genes that are phylogenetically distant from other MD8-like phages. Also, the protease and capsid of phages φ2 and TC7 are encoded by two separate genes. Conversely, the analyses of trees and alignments indicated that genes more similar to φ2 and TC7 prevail in the part of the MD8 genome encoding the lysogeny maintenance and replication genes. A block of tail proteins is closer to an MD8-like phage JBD68 and the lysis proteins are more similar to proteins of phage BR133a and others. Thus, the MD8 genome can be roughly divided into several parts, including the terminase and capsid F10-like part and the replication and lysogeny φ-like part (Figure 11).

The topologies of phylogenetic trees of the genes belonging to the same functional modules are often similar, illustrating the theory of modular evolution [72]. For example, the two terminase genes encoding the small and large subunits of terminase seem to possess a similar recent evolutionary history, as well as the genes of head-to-tail joining protein, portal protein, protease, scaffold and MCP. The phylogenetic trees demonstrate a similar topology of the branches adjacent to phage MD8, also for blocks of several tail proteins. Analyses of alignments indicated the presence of closest homologs of both TA system genes in the genome of the same phage not belonging to the MD8 group. Interestingly, the topology of the trees of the replication, lysogeny and lysis proteins is more versatile. The results of the analysis are summarised in Figure 11, where the MD8 genomic modules are associated with the phages of the MD8 group and outside this group. The phages possessing the genes encoding the homologous proteins closely related to MD8 proteins and belonging to adjacent MD8 clades of phylogenetic trees are shown in this Figure.

## 3. Discussion

### 3.1. Phage MD8 and General Problems with the Lambdoids’ Classification

The results obtained in this research indicate an MD8 genome organisation that is similar to the genome organisation of phage λ. However, only a few proteins show detectable homology with proteins of *Lambdavirus* phages. Moreover, the MD8-like phages’ proteins demonstrate different evolutionary histories. This raises questions about the possibility of building a taxonomic classification that reflects the evolutionary history of lambdoid bacteriophages. Nevertheless, the availability of meaningful taxonomic classification is essential for both theoretical and applied studies. It might be suggested that such a classification could be proposed at least for some λ-like phages. The results obtained should lead to a discussion of the efficiency of the methods used here and a possible classification algorithm.

### 3.2. Biological Properties, Genomic Organisation and Classification at the Level of Family

It seems impossible to develop a comprehensive taxonomic description of phages based on their biological properties, such as burst size, growth condition, UV-resistance, etc. The temperate lifestyle is intrinsic for very distant phage groups, and very distant phages can possess similar host specificity or latent period. Interestingly, recent findings have demonstrated that even the phage lifestyle can be altered within a taxonomically established group such as *Autographiviridae*, where the temperate phages were discovered [73,74,75,76]. Biological properties can lead to some assumptions, but, generally, they cannot be used for classification within the λ-like phage group. Meanwhile, genomic organisation is often used for a general description of the phage genome. Genomic organisation includes the list and order of genes, regulatory elements and DNA spatial structure [77,78,79]. Terminal repeats in phage genomes can also be attributed to elements of genomic structure. Because of exceptional genetic mosaicism, it is not possible to trace evolutionary relations using the whole genomes of lambdoid phages, but all the lambdoids share an overall similarity in terms of genome organisation. According to the latest 15-rank taxonomic classification of viruses proposed by the ICTV [80], the tailed bacteriophages are assigned to the order of *Caudovirales*. The next hierarchical ranks are suborder and family. It would probably be appropriate to elevate the λ-like phages to the level of family, and in this context, genome organisation could be the main criterion for belonging to the lambdoid phage family, independently of the origin of individual genes.

### 3.3. Applicability of the Whole-Genome and Proteome Comparisons

Whole-genome and proteome comparisons conducted using VIRIDIC, ANI and ViPtree showed that phage MD8 shares detectable similarities with a few dozen *Pseudomonas* phages. The results of all three methods are close, but the calculations with orthoANI gave high ANI values for taxonomically distant phages when only small fractions of compared genomes aligned. For example, the ANI value calculated with orthoANIu for a group of *Pseudomonas* Podoviruses belonging to the genus *Hollowayvirus* was 97.1%, which is higher than the calculated ANI values for all the other phages, although the length of the aligned fragment was about 400 bases only. This can lead to the wrong conclusions, especially in the case of recent horizontal transfers, so it is necessary also to check the size of aligned fragments of the genome. OrthoANIu is fast and the ANI analysis is popular, but the VIRIDIC pipeline appears to be more convenient and flexible here. The results calculated by VIRIDIC and represented by VIRIDIC’s similarity matrix are partially consistent with the results of the phylogenetic analysis of the MD8 genes. The analysis of proteome performed with ViPtree agrees with the VIRIDIC’s results but neither ViPtree nor VIRIDIC can reflect true evolutionary history, due to the different evolutionary histories of different phage proteins. Besides, the processes of gene exchange between lambdoid phages may cause the spread of intergenomic similarity values. In this case, partially overlapping clusters were observed, (as illustrated in Figure 9D). To avoid uncertainty and an excessive number of taxa, the genus threshold of λ-like phages might be lowered.

It might be argued that only a combination of various whole-genome comparison methods should be used for the clarification of relations of lambdoid phages within this group and with other phages, and possible relationships should be conferred through more thorough genomic studies. The regular threshold for delineation of bacteriophage species based on genomic similarity is 95% and for genus delineation it is 70%. We doubt the applicability of this intergenomic similarity as a single criterion, but suggest its usefulness for species delineation in particular.

### 3.4. Phylogenetic Analysis of Individual Genes and Genomic Modules

The phylogenetic studies have clearly demonstrated the dissimilar evolutionary history of MD8 proteins. This not only is a feature of λ-like and temperate phages, to different degrees, but also is relevant to other tailed bacteriophages [81].

The concatenated genes (proteins) phylogeny is often employed for the reconstruction of an evolutionary history [82], but it cannot be used when the genes have a contradictory evolutionary history [83]. However, it can assist the elucidation of the evolutionary history of modules which share the same origin. It is a fact that the lambdoid genome is a chimera, but phylogenetic analysis makes it possible to distinguish a group of genes sharing the same origin and representing the absolute or relative majority of the phage genes.

The elucidation of the evolutionary history of genetic modules can help to distinguish different phage groups, but the task of building a consistent hierarchical taxonomy is more complex. It appears that there is very little scope for using the phylogenetic relations between genomic modules as a single criterion for phage taxonomic classification, not only due to the very principle of the formation of phage genomes as a mosaic structure of genes of various origins, but also because of fast sequence drift [84].

### 3.5. Delineation of Subfamilies, Genera and Species

If genomic organisation could be the main attribute for the delineation of the phage lambdoid group as a family, then the delineation of subfamilies could be based on the taxonomy of infected hosts. The prophage genes of lambdoid phages are parts of the host chromosomes (except for plasmid phages). Interestingly, ANI calculations provide about 87% of nucleotide identity at high coverage for phage MD8 and *Pseudomonas aeruginosa* (strain BH9), 95% for phage λ and *Escherichia coli* and 73% for phage N15 and *Escherichia coli* (strain THO-003). All the MD8 proteins have homologs in *Pseudomonas* genomes. That is why it would probably be appropriate to assign all *Pseudomonas* lambdoid phages to a single subfamily.

The delineation of genera could be based on the analysis of the evolutionary history of such marker genes as MCP and terminase. As has been shown by the current research, the evolutionary history of the terminase and the major capsid protein modules can differ and the recent evolutionary history of the majority of phage genes, combined with the results of whole-genome and proteome comparison and clustering, could also be used to clarify lambdoids’ taxonomy.

**Figure 12:**
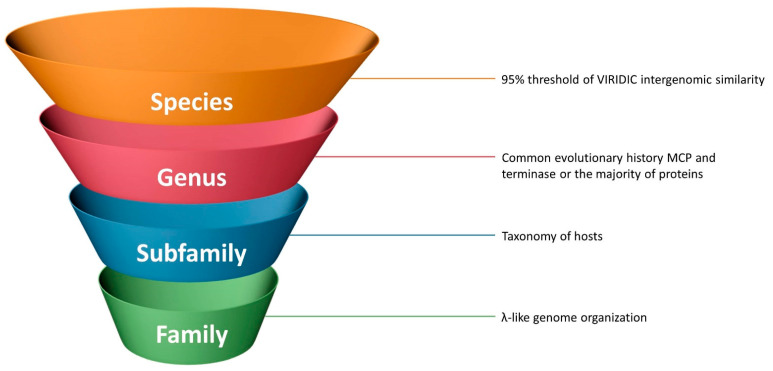
Possible criteria for the taxonomic classification of lambdoid bacteriophages.

The traditional 95% threshold for intergenomic similarity seems to be a good criterion for lambdoid species delineation, since it assumes nearly identical genome organisation and evolutionary history for the majority of phage genes and places too similar phages into clonal groups (Figure 12).

Applying this reasoning, the 22 *Pseudomonas* bacteriophages, which are MD8, F10, JBD68, PAN70, BR133a, BR144a, BR200a, BR201a, BR204a, BR213a, BR233a, BR299a, CF24b, CF28b, CF34a, CF55b, CF60a, CF67a, CF79a, CF140a, CF145a, CF165a and CF208a, could be classified as representatives of a single genus. The intergenomic similarity within the VIRIDIC cluster of these phages is lower than the genus threshold of 70%, but these phages possess similar terminase and MCP genes, which comprise the protease, scaffold protein and MCP domains, and the majority of genes of these phages have a similar recent evolutionary history. The intergenomic similarity of bacteriophages BR201a and CF3a compared to the majority of the phages listed above is higher than 70% and the majority of proteins of these two phages are very similar to the proteins of the 22 phages listed above, but their partial genome sequences lack the terminase and MCP genes. Probably, these two phages also can be assigned to the same genus as the 22 phages listed above. Phages BR204a, BR233a, BR299a, CF24b, vB_Pae_CF28b, CF140a, CF145a, CF165a and CF208a, as well as phages BR133a, CF55b and CF79a, represent races of the same two species because of the high level of genome similarity (the VIRIDIC intergenomic similarity is higher 95%).

Phages φ2 and TC7 could constitute another related genus of the subfamily of *Pseudomonas* λ-like phages of the lambdoid viral family. The VIRIDIC intergenomic similarity of these two phages is 70.7% and these bacteriophages possess similar terminases and MCPs, which are distantly related with terminases and MCPs of the rest of the MD8-like phages.

## 4. Materials and Methods

### 4.1. Phage Propagation and Purification

*Pseudomonas aeruginosa* laboratory strain PAO1, kindly provided by Prof. Victor N. Krylov, was used as a host for phage propagation. A water sample from Lake Baikal (3 mL) was supplemented with 1 mL of a 4-x sterile nutrient medium (LB), and 40 μL of an overnight PAO1 culture was added. The culture was incubated for 18 h at 18 °C. Chloroform was added to a final concentration of 0.5% (*v*/*v*) for 4 h at 4 °C. The suspension was then centrifuged at 7000× *g* for 20 min. The presence of bacteriophages in the supernatant was assessed by the appearance of plaques on the bacterial lawn of the host strain. The titer was determined by means of the double agar layer method using LB with 0.7% top agar [85]. Plaques formed were counted after 18–24 h of incubation at 25 °C. In addition to the method described above, for the isolation of phages, the authors tested the technique of sorption of viruses from a large volume of water on a Hi-TrapQ ion-exchange resin (GE Healthcare, Chicago, IL, USA). Before being loaded onto the column, a water sample (about 400 mL) was filtered through a membrane filter (0.22 µm, Millipore, Burlington, MA, USA) and buffered at pH 7.5 adding 1 M Tris-HCl stock to a 50 mM concentration. Phage particles bound to the resin were eluted from the column with 50 mM Tris-HCl pH 7.5, 4 M NaCl and concentrated using Centricon 100 (Amicon, Northfield, OH, USA). Aliquots of concentrated eluate (0.1 mL) were applied to the top agar with *P. aeruginosa* PAO1.

To propagate the phage from a single plaque, a liquid culture of *P. aeruginosa* PAO1 was incubated at 37 °C until the end of lysis, followed by the addition of chloroform. The cell debris was pelleted by centrifugation at 7000× *g* for 20 min. Phage lysate was precipitated with the addition of polyethene glycol 8000 (10%) and NaCl (0.6%) at 4 °C overnight, followed by centrifugation (8000× *g*, 20 min, 4 °C), and the pellet was resuspended in SM buffer (50 mM Tris-HCl (pH 7.5), 100 mM NaCl, 8 mM MgSO_4_, 0.01% gelatin).

After 20 min incubation on ice, the mixture was centrifuged again (12,000× *g* for 20 min at 4 °C), and the phage pellet was collected [86]. The resulting preparation was purified in an equilibrium caesium chloride gradient by centrifugation for 2 h at 22,000× *g* (Beckman SW41 Ti rotor, Beckman Coulter Inc., Brea, CA, USA). A band with bright opalescence was collected and dialysed overnight against 0.01 M Tris-HCl (pH 7.5), 0.01 M MgSO_4_, 0.15 M NaCl (4 °C).

### 4.2. Phage Biology Experiments

To determine the time of adsorption, host cells were grown to OD600 = 0.3 at 28 °C and a phage suspension with a multiplicity of infection (MOI) = 0.001 was added. After 1, 2, 3, 4, 5, 8, 10, 15 and 20 min, 100 μL samples were taken and 850 μL of SM buffer and 50 μL of chloroform were added. The samples were centrifuged and the phage titer was determined by the plaque assay method at different time intervals.

For a one-step growth experiment, 50 mL of host cells (OD600 nm = 0.3) were centrifuged (7000× *g*, 20 min, 4 °C) and resuspended in 0.5 mL of warmed LB medium. A bacteriophage with an MOI of 0.01 was added. The mixture was incubated to adsorb the bacteriophage for 5 min at 37 °C. Then, it was centrifuged at 10,000× *g* for 2 min to remove non-adsorbed phage particles and the sediment was resuspended in 20 mL of LB medium. During a 2 h incubation at 37 °C, samples were taken at 5 min intervals and titrated immediately.

To analyse temperature sensitivity, a phage suspension (1 × 10^3^ pfu/mL) was incubated at 0 °C, 4 °C, 20 °C, 37 °C, 42 °C and 50 °C for 1 h, after which the phage titer in the samples was determined. For the analysis of pH stability, the phage suspension was inoculated to the same concentration in a series of tubes containing fresh LB broth at pH 2.0, 5.0, 7.0, 9.0 and 12.0, as described in [87], and incubated at room temperature; aliquots for titration were taken after 24 h of incubation.

The effect of salinity was studied by adding phage to a concentration of 10^3^ pfu/mL to NaCl solutions with a concentration of 0.01, 0.1, 1 and 5 M. The phage titer was determined on double agar after 24 h. To test the effect of chloroform on the stability of phages, 200 μL of chloroform was added to 5 mL of phage suspension, incubated for 1 h at room temperature and aliquots were plated on double agar. To assess UV stability, phage suspensions at the same concentration were exposed under a UV lamp (Lamsystems, Moscow, Russia) for 5 and 10 min and the titer was determined as described above.

To analyse the host range, the MD8 phage was tested against 125 clinical and environmental strains of *P. aeruginosa* on double-layer agar [85]. Phage suspension 10^9^ pfu/mL was applied to the surface of soft agar (0.7% *w*/*v*) and incubated at 37 °C for 18–24 h. The appearance of a clear zone indicated the presence of lytic activity.

All procedures were repeated in triplicate and the results were averaged.

### 4.3. Electron Microscopy

The morphology of the MD8 phage was studied using transmission electron microscopy (TEM) [88]. Concentrated purified phage samples were placed on grids and stained with 1% aqueous uranyl acetate (pH 4.0). Images were obtained using a Hitachi H-300TM electron microscope (Hitachi Ltd., Tokyo, Japan).

### 4.4. Phage Sequencing and Annotation

Phage DNA was fragmented with medium-size fragments of about 600 bp in a microTUBE Adaptive Focused Acoustics (AFA) fibry snap-cap tube using a Covaris S2 instrument (Covaris, Woburn, MA, USA). The DNA library was constructed using the dual-index NEBNext multiplex oligos (New England Biolabs, Ipswich, MA, USA) and the NEBNext Ultra II DNA library prep kit for Illumina (New England Biolabs). The library was size-selected on a Blue Pippin 1.5% agarose DNA gel (Sage Science, Beverly, MA, USA) with size-selection settings of 550–1000 bp. This DNA library was sequenced with reagent kit version 3 (600-cycle) on a MiSeq platform (Illumina) at the SB RAS Genomics Core Facility (ICBFM SB RAS, Novosibirsk, Russia). The entire genome was assembled de novo using SPAdes software version 3.15, with default parameters [89]. The genome of *Pseudomonas* phage MD8 has been deposited in the NCBI GenBank under Accession number KX198612.

The MD8 genome was annotated with the assistance of Prokka [90], using custom databases made with BLAST tools [91], the RVDB database [92] and Prokka default databases. Open reading frames (ORFs) were predicted with Prodigal 2.6.1 [93], Glimmer 3.02b [94] and Geneious Prime 2021.1 [95] and manually validated and curated.

The prediction of functions of the encoded protein was made through a homology search and an HMM-motif comparison. The homology search was performed by BLAST, using the NCBI non-redundant (nr/nt) database and custom databases made with BLAST using GenBank phage sequences, and the functions were assigned by comparison with known homologs. The HHM-motif search was conducted with Phyre2 [96] and the HHpred server (PDB_mmCIF70, SCOPe70_2.07, ECOD_ECOD_F70, and UniProt-SwissProt-viral70 databases) [97,98], and the functions were assigned through comparison with similar proteins with the threshold of 95% Phyre2 confidence or HHpred probability. The presence of tRNA coding regions was checked using tRNAscan-SE [99] and ARAGORN [100]. The genetic maps were visualised in Geneious Prime 2021.1.

### 4.5. Intergenomic Comparison and Proteome Analysis

Genomic sequences of bacteriophages were downloaded from GenBank. The intergenomic comparison was made with the orthoANIu pipeline employing the USEARCH algorithm for the similarity search [101,102,103] and with the VIRIDIC tool designed for finding intergenomic similarities of prokaryote-infecting viruses [31]. The proteome comparison, clustering and dendrogram plotting were made using the ViPtree server [70]. The intergenomic comparison diagrams were made with the Easyfig [104] program, using TBLASTX for finding similarities between the encoded proteins.

### 4.6. Phylogenetic Analysis

The alignments were made with MAFFT 7.48 with default settings using the L-INS-i algorithm [105,106]. The alignments were trimmed manually or with trimAL [107] with gappyout settings if needed. The best protein model was found with ModelTest-NG [108,109]. The final phylogenetic trees were constructed with MRBAYES [110,111]. The topological accuracy of the MRBAYES tree was estimated using values of the average standard deviation of split frequencies and posterior probability. In cases where the number of homologous sequences was excessive for the analysis, the representative sequences were chosen using intermediate trees. These sequences represented all the adjacent clades and other main clades. To speed up the calculations, intermediate trees were constructed with FastTree 2.1.11 [112,113] using Whelan and Goldman’s 2001 model and with RAxML 8.2.1 [114,115] using the CAT LG [116] model and rapid bootstrapping and a search for the best-scoring tree algorithm. The robustness of the RAxML trees was assessed by bootstrapping (100 and higher). The trees were visualised using Geneious Prime 2021.1.

### 4.7. Analysis and Modelling of Protein Structure

Secondary structures of phage proteins were predicted using the HHpred [97,98] and Phyre2 [96] servers. Spatial structures were obtained using Phyre2 and visualised in UCSF Chimera [117]. Structural alignments were made with Chimera.

## 5. Conclusions

*Pseudomonas* bacteriophage MD8 represents a group of temperate phages that feature the lambdoid organisation of the genome. The genomes of MD8-like phages encode relatively distant from phage λ proteins, but many of those proteins possess functionality analogous to the phage λ proteins. The MD8 genome has a mosaic origin, hindering the elucidation of true evolutionary history and the assignment of a taxonomic position to this phage. However, based on the evolutionary history of the majority of the proteins of MD8-like phages and intergenomic comparisons, it is proposed that the group comprising 26 phages be assigned to two novel genera.

## Figures and Tables

**Table 1:** ANI values compared to *Pseudomonas* phage MD8 for all of the 14,983 GenBank phage genomes demonstrating aligned length with MD8 of more than 10%.

NCBI Accession	ANI	Average Aligned Length	Genome Length	Phage
KX198612	100.00	42,840	42,840	*Pseudomonas* phage MD8
MK510975	96.19	10,023	39,780	*Pseudomonas* phage vB_Pae_CF145a
MK510976	95.86	11,062	39,780	*Pseudomonas* phage vB_Pae_CF165a
MK510964	95.08	10,158	39,780	*Pseudomonas* phage vB_Pae_CF28b
MK510968	94.95	13,160	41,820	*Pseudomonas* phage vB_Pae_CF55b
MK510969	94.51	9288	34,680	*Pseudomonas* phage vB_Pae_CF60a
MK510986	94.08	15,886	30,600	*Pseudomonas* phage vB_Pae_BR213a
MK510988	93.96	14,820	39,780	*Pseudomonas* phage vB_Pae_BR299a
MK510962	93.92	7927	33,660	*Pseudomonas* phage vB_Pae_CF3a
MK510990	93.91	11,921	40,800	*Pseudomonas* phage vB_Pae_BR133a
MK510963	93.88	16,786	39,780	*Pseudomonas* phage vB_Pae_CF24b
MK510970	93.68	14,779	34,680	*Pseudomonas* phage vB_Pae_CF67a
MK510974	93.68	17,260	39,780	*Pseudomonas* phage vB_Pae_CF140a
MK510987	93.58	17,309	39,780	*Pseudomonas* phage vB_Pae_BR233a
MK510971	93.57	14,897	40,800	*Pseudomonas* phage vB_Pae_CF79a
MK510985	93.56	17,306	39,780	*Pseudomonas* phage vB_Pae_BR204a
MK510978	93.25	15,465	39,780	*Pseudomonas* phage vB_Pae_CF208a
KT887558	93.11	11,613	41,820	*Pseudomonas* phage φ2
MK510965	93.07	15,680	40,800	*Pseudomonas* phage vB_Pae_CF34a
MK510984	92.96	10,307	37,740	*Pseudomonas* phage vB_Pae_BR200a
MK510992	92.93	12,157	39,780	*Pseudomonas* phage vB_Pae_BR144a
MG707188	92.39	10,879	42,840	*Pseudomonas* phage TC7
KY707339	92.35	17,040	39,780	*Pseudomonas* phage JBD68
KJ959591	91.60	15,111	38,760	*Pseudomonas* phage PAN70
DQ163912	91.59	16,514	38,760	*Pseudomonas* phage F10
MK510993	89.11	5095	37,740	*Pseudomonas* phage vB_Pae_BR201a

## Supplementary Materials

The Supplementary Materials are available online at https://www.mdpi.com/article/10.3390/ijms221910350/s1.

## References

[1] Esther M. Zimmer Lederberg Memorial Website. http://www.estherlederberg.com/home.html.

[2] Lederberg E.M., Lederberg J. (1953). Genetic studies of lysogenicity in *Escherichia coli*. Genetics.

[3] Weigle J.J. (1953). Induction of Mutations in a Bacterial Virus. Proc. Natl. Acad. Sci. USA.

[4] Jacob F., Wollman E.L. (1953). Induction of Phage Development in Lysogenic Bacteria. Cold Spring Harb. Symp. Quant. Biol..

[5] Kaiser A.D. (1955). A genetic study of the temperate coliphage λ. Virology.

[6] Campbell A. (1961). Sensitive mutants of bacteriophage λ. Virology.

[7] Casjens S.R., Hendrix R.W. (2015). Bacteriophage lambda: Early pioneer and still relevant. Virology.

[8] Dove W.F. (1968). The Genetics of the Lambdoid Phages. Annu. Rev. Genet..

[9] Campbell A. (1994). Comparative Molecular Biology of Lambdoid Phages. Annu. Rev. Microbiol..

[10] Kameyama L., Fernandez L., Calderon J., Ortiz-Rojas A., Patterson T.A. (1999). Characterization of Wild Lambdoid Bacteriophages: Detection of a Wide Distribution of Phage Immunity Groups and Identification of a Nus-Dependent, Nonlambdoid Phage Group. Virology.

[11] Campbell A., Botstein D. (1983). Evolution of the Lambdoid Phages. Cold Spring Harb. Monogr. Arch..

[12] Juhala R.J., E Ford M., Duda R., Youlton A., Hatfull G.F., Hendrix R.W. (2000). Genomic sequences of bacteriophages HK97 and HK022: Pervasive genetic mosaicism in the lambdoid bacteriophages. J. Mol. Biol..

[13] Brüssow H., Hendrix R.W. (2002). Phage Genomics: Small Is Beautiful. Cell.

[14] Clark A.J., Inwood W., Cloutier T., Dhillon T. (2001). Nucleotide sequence of coliphage HK620 and the evolution of lambdoid phages. J. Mol. Biol..

[15] Campbell A., Schneider S.J., Song B. (1992). Lambdoid phages as elements of bacterial genomes (integrase/phage21/Escherichia coli K-12/icd gene). Genetica.

[16] Brüssow H., Canchaya C., Hardt W.-D. (2004). Phages and the Evolution of Bacterial Pathogens: From Genomic Rearrangements to Lysogenic Conversion. Microbiol. Mol. Biol. Rev..

[17] Hofer U. (2020). Stop that plasmid. Nat. Rev. Genet..

[18] Drucker V.V., Dutova N.V. (2006). Study of the morphological diversity of bacteriophages in Lake Baikal. Dokl. Biol. Sci..

[19] Drucker V.V., Potapov S.A., Gorshkova A.S., Belykh O.I. (2020). Bacteriophages of Lake Baikal.

[20] Matsumoto T., Fujita M. (2018). Chronic Pseudomonas aeruginosa Infection as the Pathogenesis of Chronic Obstructive Pulmonary Disease. Bacterial Pathogenesis and Antibacterial Control.

[21] Crull M.R., Somayaji R., Ramos K.J., Caldwell E., Mayer-Hamblett N., Aitken M.L., Nichols D.P., Rowhani-Rahbar A., Goss C.H. (2018). Changing Rates of Chronic Pseudomonas aeruginosa Infections in Cystic Fibrosis: A Population-Based Cohort Study. Clin. Infect. Dis..

[22] Serra R., Grande R., Butrico L., Rossi A., Settimio U.F., Caroleo B., Amato B., Gallelli L., de Franciscis S. (2015). Chronic wound infections: The role of *Pseudomonas aeruginosa* and *Staphylococcus aureus*. Expert Rev. Anti-Infect. Ther..

[23] Kwan T., Liu J., DuBow M., Gros P., Pelletier J. (2006). Comparative Genomic Analysis of 18 Pseudomonas aeruginosa Bacteriophages. J. Bacteriol..

[24] Yu X., Xu J., Gu Y., Zhang R., Zhu Y., Liu X. (2020). Molecular Characterization and Comparative Genomic Analysis of vB_PaeP_YA3, a Novel Temperate Bacteriophage of *Pseudomonas aeruginosa*. Front. Microbiol..

[25] International Committee on Taxonomy of Viruses (ICTV). https://talk.ictvonline.org/?Redirected=true.

[26] Shapiro J.W., Putonti C. (2018). Gene Co-occurrence Networks Reflect Bacteriophage Ecology and Evolution. mBio.

[27] Chibani C.M., Farr A., Klama S., Dietrich S., Liesegang H. (2019). Classifying the Unclassified: A Phage Classification Method. Viruses.

[28] Holguín A.V., Rangel G., Clavijo V., Prada C., Mantilla M., Gomez M.C., Kutter E., Taylor C., Fineran P.C., Barrios A.F.G. (2015). Phage ΦPan70, a Putative Temperate Phage, Controls Pseudomonas aeruginosa in Planktonic, Biofilm and Burn Mouse Model Assays. Viruses.

[29] Tariq M.A., Everest F.L.C., Cowley L., Wright R., Holt G.S., Ingram H., Duignan L.A., Nelson A., Lanyon C.V., Perry A. (2019). Temperate Bacteriophages from Chronic Pseudomonas aeruginosa Lung Infections Show Disease-Specific Changes in Host Range and Modulate Antimicrobial Susceptibility. mSystems.

[30] Van Belkum A., Soriaga L.B., LaFave M.C., Akella S., Veyrieras J.-B., Barbu E.M., Shortridge D., Blanc B., Hannum G., Zambardi G. (2015). Phylogenetic Distribution of CRISPR-Cas Systems in Antibiotic-Resistant Pseudomonas aeruginosa. mBio.

[31] Moraru C., Varsani A., Kropinski A. (2020). VIRIDIC—A Novel Tool to Calculate the Intergenomic Similarities of Prokaryote-Infecting Viruses. Viruses.

[32] Smith K.C., Castro-Nallar E., Fisher J.N., Breakwell D.P., Grose J.H., Burnett S.H. (2013). Phage cluster relationships identified through single gene analysis. BMC Genom..

[33] King J., Casjens S. (1974). Catalytic head assembling protein in virus morphogenesis. Nat. Cell Biol..

[34] Duda R.L., Oh B., Hendrix R.W. (2013). Functional Domains of the HK97 Capsid Maturation Protease and the Mechanisms of Protein Encapsidation. J. Mol. Biol..

[35] Fokine A., Rossmann M.G. (2016). Common Evolutionary Origin of Procapsid Proteases, Phage Tail Tubes, and Tubes of Bacterial Type VI Secretion Systems. Structure.

[36] Duda R.L., Hempel J., Michel H., Shabanowitz J., Hunt D., Hendrix R.W. (1995). Structural transitions during bacteriophage HK97 head assembly. J. Mol. Biol..

[37] Medina E., Wieczorek D., Medina E.M., Yang Q., Feiss M., Catalano C.E. (2010). Assembly and Maturation of the Bacteriophage Lambda Procapsid: GpC Is the Viral Protease. J. Mol. Biol..

[38] Chang J.R., Spilman M.S., Rodenburg C.M., Dokland T. (2009). Functional domains of the bacteriophage P2 scaffolding protein: Identification of residues involved in assembly and protease activity. Virology.

[39] Chen Z., Sun L., Zhang Z., Fokine A., Padilla-Sanchez V., Hanein D., Jiang W., Rossmann M.G., Rao V.B. (2017). Cryo-EM structure of the bacteriophage T4 isometric head at 3.3-Å resolution and its relevance to the assembly of icosahedral viruses. Proc. Natl. Acad. Sci. USA.

[40] Guo F., Liu Z., Fang P.-A., Zhang Q., Wright E.T., Wu W., Zhang C., Vago F., Ren Y., Jakana J. (2014). Capsid expansion mechanism of bacteriophage T7 revealed by multistate atomic models derived from cryo-EM reconstructions. Proc. Natl. Acad. Sci. USA.

[41] Casjens S., King J. (1974). P22 morphogenesis I: Catalytic scaffolding protein in capsid assembly. J. Supramol. Struct..

[42] Nelson R.A., E Reilly B., Anderson D.L. (1976). Morphogenesis of bacteriophage phi 29 of Bacillus subtilis: Preliminary isolation and characterization of intermediate particles of the assembly pathway. J. Virol..

[43] Shaw J.E., Murialdo H. (1980). Morphogenetic genes C and Nu3 overlap in bacteriophage λ. Nature.

[44] Zylicz M., Liberek K., Wawrzynow A., Georgopoulos C. (1998). Formation of the Preprimosome Protects λ O from RNA Transcription-Dependent Proteolysis by ClpP/ClpX. Proc. Natl. Acad. Sci. USA.

[45] Roberts R., McMacken R. (1983). The bacteriopbage λ O replication protein: Isolation and characterization of the amplified initiator. Nucleic Acids Res..

[46] Hayes S., Erker C., Horbay M.A., Marciniuk K., Wang W., Hayes C. (2013). Phage Lambda p Protein: Trans-Activation, Inhibition Phenotypes and Their Suppression. Viruses.

[47] Van der Wilk F., Dullemans A., Verbeek M., Heuvel J.F.V.D. (1999). Isolation and Characterization of APSE-1, a Bacteriophage Infecting the Secondary Endosymbiont of *Acyrthosiphon pisum*. Virology.

[48] Ravin V., Ravin N., Casjens S., Ford M.E., Hatfull G.F., Hendrix R.W. (2000). Genomic sequence and analysis of the atypical temperate bacteriophage N15. J. Mol. Biol..

[49] Tarkowski T.A., Mooney D., Thomason L.C., Stahl F.W. (2002). Gene Products Encoded in the NinR Region of Phage λ Participate in Red-Mediated Recombination. Genes Cells.

[50] Hollifield W.C., Kaplan E.N., Huang H.V. (1987). Efficient recABC-dependent, homologous recombination between coliphage lambda and plasmids requires a phage ninR region gene. Mol. Genet. Genom..

[51] Kaiser A.D. (1957). Mutations in a temperate bacteriophage affecting its ability to lysogenize *Escherichia coli*. Virology.

[52] Johnson A., Meyer B.J., Ptashne M. (1978). Mechanism of action of the cro protein of bacteriophage lambda. Proc. Natl. Acad. Sci. USA.

[53] García P., Ladero V., Alonso J.C., Suárez J.E. (1999). Cooperative Interaction of CI Protein Regulates Lysogeny of Lactobacillus casei by Bacteriophage A2. J. Virol..

[54] Kornitzer D., Altuvia S., Oppenheim A.B. (1991). The activity of the CIII regulator of lambdoid bacteriophages resides within a 24-amino acid protein domain. Proc. Natl. Acad. Sci. USA.

[55] Kobiler O., Rokney A., Oppenheim A.B. (2007). Phage Lambda CIII: A Protease Inhibitor Regulating the Lysis-Lysogeny Decision. PLoS ONE.

[56] Hayes F., Austin S.J. (1993). Specificity determinants of the P1 and P7 plasmid centromere analogs. Proc. Natl. Acad. Sci. USA.

[57] Letarov A.V., Kulikov E. (2017). Adsorption of bacteriophages on bacterial cells. Biochemistry.

[58] Dupont K., Vogensen F.K., Neve H., Bresciani J., Josephsen J. (2004). Identification of the Receptor-Binding Protein in 936-Species Lactococcal Bacteriophages. Appl. Environ. Microbiol..

[59] Fokine A., Islam M.Z., Zhang Z., Bowman V.D., Rao V.B., Rossmann M.G. (2011). Structure of the Three N-Terminal Immunoglobulin Domains of the Highly Immunogenic Outer Capsid Protein from a T4-Like Bacteriophage. J. Virol..

[60] Werts C., Michel V., Hofnung M., Charbit A. (1994). Adsorption of bacteriophage lambda on the LamB protein of Escherichia coli K-12: Point mutations in gene J of lambda responsible for extended host range. J. Bacteriol..

[61] Randall-Hazelbauer L., Schwartz M. (1973). Isolation of the Bacteriophage Lambda Receptor from *Escherichia coli*. J. Bacteriol..

[62] Hendrix R., Duda R. (1992). Bacteriophage lambda PaPa: Not the mother of all lambda phages. Science.

[63] Berry J., Rajaure M., Pang T., Young R. (2012). The Spanin Complex Is Essential for Lambda Lysis. J. Bacteriol..

[64] Young R. (2014). Phage lysis: Three steps, three choices, one outcome. J. Microbiol..

[65] Evseev P., Lukianova A., Shneider M., Korzhenkov A., Bugaeva E., Kabanova A., Miroshnikov K., Kulikov E., Toshchakov S., Ignatov A. (2020). Origin and Evolution of *Studiervirinae* Bacteriophages Infecting *Pectobacterium*: Horizontal Transfer Assists Adaptation to New Niches. Microorganisms.

[66] Shneider M.M., Lukianova A.A., Evseev P.V., Shpirt A.M., Kabilov M.R., Tokmakova A.D., Miroshnikov K.K., Obraztsova E.A., Baturina O.A., Shashkov A.S. (2020). Autographivirinae Bacteriophage Arno 160 Infects Pectobacterium carotovorum via Depolymerization of the Bacterial O-Polysaccharide. Int. J. Mol. Sci..

[67] Kędzierska B., Hayes F. (2016). Emerging Roles of Toxin-Antitoxin Modules in Bacterial Pathogenesis. Molecules.

[68] Liu Y., Gao Z., Liu G., Geng Z., Dong Y., Zhang H. (2020). Structural Insights Into the Transcriptional Regulation of HigBA Toxin–Antitoxin System by Antitoxin HigA in Pseudomonas Aeruginosa. Front. Microbiol..

[69] Craig L., Taylor R.K., E Pique M., Adair B.D., Arvai A.S., Singh M., Lloyd S.J., Shin D.S., Getzoff E.D., Yeager M. (2003). Type IV Pilin Structure and Assembly. Mol. Cell.

[70] Nishimura Y., Yoshida T., Kuronishi M., Uehara H., Ogata H., Goto S. (2017). ViPTree: The viral proteomic tree server. Bioinformatics.

[71] Latino L., Essoh C., Blouin Y., Thien H.V., Pourcel C. (2014). A novel *Pseudomonas aeruginosa* Bacteriophage, Ab31, a Chimera Formed from Temperate Phage PAJU2 and *P. putida* Lytic Phage AF: Characteristics and Mechanism of Bacterial Resistance. PLoS ONE.

[72] Botstein D. (1980). A Theory of Modular Evolution for bacteriophages. Ann. N. Y. Acad. Sci..

[73] Labrie S., Frois-Moniz K., Osburne M., Kelly L., Roggensack S.E., Sullivan M.B., Gearin G., Zeng Q., Fitzgerald M., Henn M.R. (2013). Genomes of marine cyanopodoviruses reveal multiple origins of diversity. Environ. Microbiol..

[74] Huang S., Zhang S., Jiao N., Chen F. (2015). Comparative Genomic and Phylogenomic Analyses Reveal a Conserved Core Genome Shared by Estuarine and Oceanic Cyanopodoviruses. PLoS ONE.

[75] Mizuno C.M., Rodriguez-Valera F., Kimes N.E., Ghai R. (2013). Expanding the Marine Virosphere Using Metagenomics. PLoS Genet..

[76] Zhao Y., Qin F., Zhang R., Giovannoni S.J., Zhang Z., Sun J., Du S., Rensing C. (2019). Pelagiphages in thePodoviridaefamily integrate into host genomes. Environ. Microbiol..

[77] Glusman G., Smit A.F.A. (2009). Genome Organization. Encyclopedia of Complexity and Systems Science.

[78] Zheng H., Xie W. (2019). The role of 3D genome organization in development and cell differentiation. Nat. Rev. Mol. Cell Biol..

[79] Misteli T. (2007). Beyond the Sequence: Cellular Organization of Genome Function. Cell.

[80] International Committee on Taxonomy of Viruses Executive Committee (2020). The new scope of virus taxonomy: Partitioning the virosphere into 15 hierarchical ranks. Nat. Microbiol..

[81] Kang H.S., McNair K., Cuevas D., Bailey B., Segall A., Edwards R. (2017). Prophage Genomics Reveals Patterns in Phage Genome Organization and Replication. bioRxiv.

[82] Low S.J., Džunková M., Chaumeil P.-A., Parks D.H., Hugenholtz P. (2019). Evaluation of a concatenated protein phylogeny for classification of tailed double-stranded DNA viruses belonging to the order Caudovirales. Nat. Microbiol..

[83] Gontcharov A.A., Marin B., Melkonian M. (2003). Are Combined Analyses Better Than Single Gene Phylogenies? A Case Study Using SSU rDNA and rbcL Sequence Comparisons in the Zygnematophyceae (Streptophyta). Mol. Biol. Evol..

[84] Glazko G., Makarenkov V., Liu J., Mushegian A. (2007). Evolutionary history of bacteriophages with double-stranded DNA genomes. Biol. Direct.

[85] Serwer P., Hayes S.J., A Thomas J., Hardies S.C. (2007). Propagating the missing bacteriophages: A large bacteriophage in a new class. Virol. J..

[86] Colombet J., Robin A., Lavie L., Bettarel Y., Cauchie H., Sime-Ngando T. (2007). Virioplankton ‘pegylation’: Use of PEG (polyethylene glycol) to concentrate and purify viruses in pelagic ecosystems. J. Microbiol. Methods.

[87] Czajkowski R., Ozymko Z., Lojkowska E. (2014). Isolation and characterization of novel soilborne lytic bacteriophages infectingDickeyaspp. biovar 3 (‘D. solani’). Plant Pathol..

[88] Brenner S., Horne R. (1959). A negative staining method for high resolution electron microscopy of viruses. Biochim. Biophys. Acta (BBA)-Bioenerg..

[89] Bankevich A., Nurk S., Antipov D., Gurevich A.A., Dvorkin M., Kulikov A.S., Lesin V.M., Nikolenko S.I., Pham S., Prjibelski A.D. (2012). SPAdes: A New Genome Assembly Algorithm and Its Applications to Single-Cell Sequencing. J. Comput. Biol..

[90] Seemann T. (2014). Prokka: Rapid Prokaryotic Genome Annotation. Bioinformatics.

[91] Altschul S.F., Gish W., Miller W., Myers E.W., Lipman D.J. (1990). Basic local alignment search tool. J. Mol. Biol..

[92] Goodacre N., Aljanahi A., Nandakumar S., Mikailov M., Khan A.S. (2018). A Reference Viral Database (RVDB) To Enhance Bioinformatics Analysis of High-Throughput Sequencing for Novel Virus Detection. mSphere.

[93] Hyatt D., Chen G.-L., Locascio P.F., Land M.L., Larimer F.W., Hauser L.J. (2010). Prodigal: Prokaryotic gene recognition and translation initiation site identification. BMC Bioinform..

[94] Delcher A.L. (1999). Improved microbial gene identification with GLIMMER. Nucleic Acids Res..

[95] Geneious Prime. http://www.geneious.com/.

[96] Kelley L.A., Mezulis S., Yates C.M., Wass M.N., Sternberg M.J.E. (2015). The Phyre2 web portal for protein modeling, prediction and analysis. Nat. Protoc..

[97] Zimmermann L., Stephens A., Nam S.-Z., Rau D., Kübler J., Lozajic M., Gabler F., Söding J., Lupas A.N., Alva V. (2018). A Completely Reimplemented MPI Bioinformatics Toolkit with a New HHpred Server at its Core. J. Mol. Biol..

[98] Söding J., Biegert A., Lupas A.N. (2005). The HHpred interactive server for protein homology detection and structure prediction. Nucleic Acids Res..

[99] Schattner P., Brooks A.N., Lowe T.M. (2005). The tRNAscan-SE, snoscan and snoGPS web servers for the detection of tRNAs and snoRNAs. Nucleic Acids Res..

[100] Laslett D., Canback B. (2004). ARAGORN, a program to detect tRNA genes and tmRNA genes in nucleotide sequences. Nucleic Acids Res..

[101] Edgar R.C. (2010). Search and clustering orders of magnitude faster than BLAST. Bioinformatics.

[102] Lee I., Kim Y.O., Park S.-C., Chun J. (2016). OrthoANI: An improved algorithm and software for calculating average nucleotide identity. Int. J. Syst. Evol. Microbiol..

[103] Yoon S.-H., Ha S.-M., Lim J., Kwon S., Chun J. (2017). A large-scale evaluation of algorithms to calculate average nucleotide identity. Antonie Van Leeuwenhoek.

[104] Sullivan M.J., Petty N., Beatson S.A. (2011). Easyfig: A genome comparison visualizer. Bioinformatics.

[105] Katoh K., Misawa K., Kuma K., Miyata T. (2002). MAFFT: A novel method for rapid multiple sequence alignment based on fast Fourier transform. Nucleic Acids Res..

[106] Katoh K., Standley D.M. (2013). MAFFT Multiple Sequence Alignment Software Version 7: Improvements in Performance and Usability. Mol. Biol. Evol..

[107] Capella-Gutierrez S., Silla-Martinez J.M., Gabaldon T. (2009). trimAl: A tool for automated alignment trimming in large-scale phylogenetic analyses. Bioinformatics.

[108] Darriba D., Posada D., Kozlov A.M., Stamatakis A., Morel B., Flouri T. (2020). ModelTest-NG: A New and Scalable Tool for the Selection of DNA and Protein Evolutionary Models. Mol. Biol. Evol..

[109] Flouri T., Izquierdo-Carrasco F., Darriba D., Aberer A., Nguyen L.-T., Minh B.Q., Von Haeseler A., Stamatakis A. (2015). The Phylogenetic Likelihood Library. Syst. Biol..

[110] Ronquist F., Huelsenbeck J.P. (2003). MrBayes 3: Bayesian phylogenetic inference under mixed models. Bioinformatics.

[111] Huelsenbeck J.P., Ronquist F. (2001). MRBAYES: Bayesian inference of phylogenetic trees. Bioinformatics.

[112] Price M.N., Dehal P.S., Arkin A.P. (2009). FastTree: Computing Large Minimum Evolution Trees with Profiles instead of a Distance Matrix. Mol. Biol. Evol..

[113] Price M.N., Dehal P.S., Arkin A.P. (2010). FastTree 2–Approximately Maximum-Likelihood Trees for Large Alignments. PLoS ONE.

[114] Stamatakis A. (2006). RAxML-VI-HPC: Maximum likelihood-based phylogenetic analyses with thousands of taxa and mixed models. Bioinformatics.

[115] Stamatakis A. (2014). RAxML version 8: A tool for phylogenetic analysis and post-analysis of large phylogenies. Bioinformatics.

[116] Le S.Q., Gascuel O. (2008). An Improved General Amino Acid Replacement Matrix. Mol. Biol. Evol..

[117] Pettersen E.F., Goddard T.D., Huang C.C., Couch G.S., Greenblatt D.M., Meng E.C., Ferrin T.E. (2004). UCSF Chimera-A visualization system for exploratory research and analysis. J. Comput. Chem..

