# Supplementary figures and images for: Pseudomonas Phage MD8: Genetic Mosaicism and Challenges of Taxonomic Classification of Lambdoid Bacteriophages
Source: Int J Mol Sci. 2021 Sep 26;22(19):10350. doi: 10.3390/ijms221910350 (PMC8508860; doi:10.3390/ijms221910350)

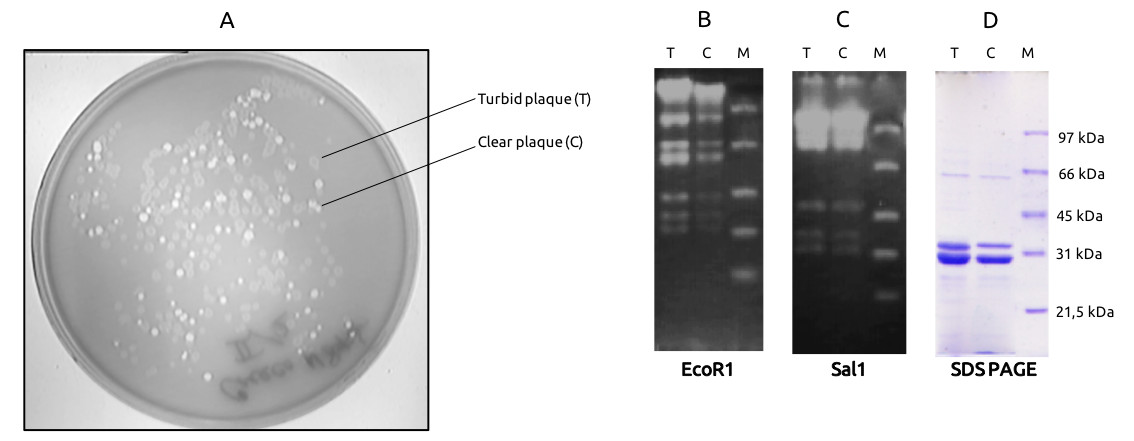

Supplement: Supplementary file 1 [file ijms-22-10350-s001.zip › SF1_cropped.jpg]

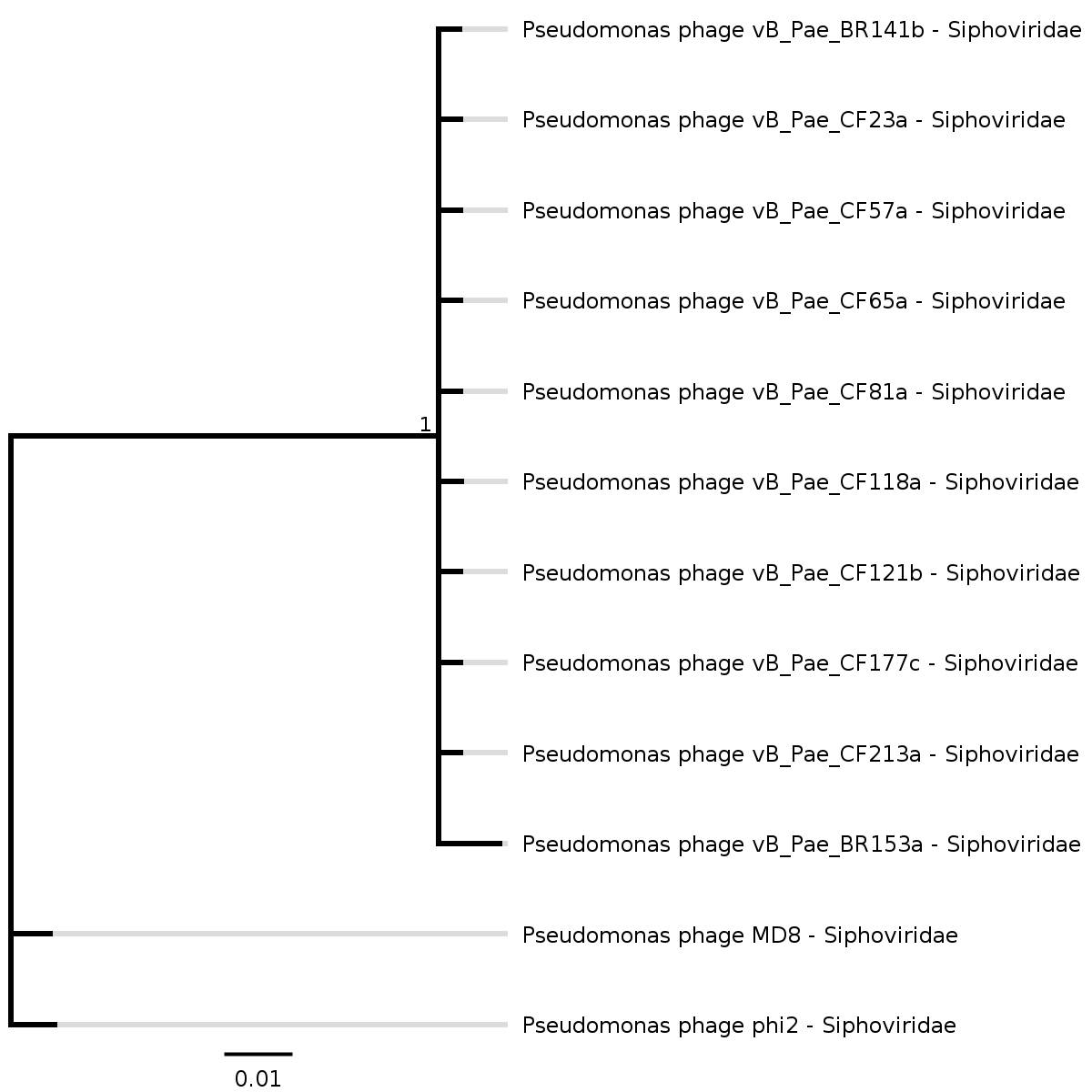

Supplement: Supplementary file 1 [file ijms-22-10350-s001.zip › SF10_gp54_repressor protein Cro_0.jpg]

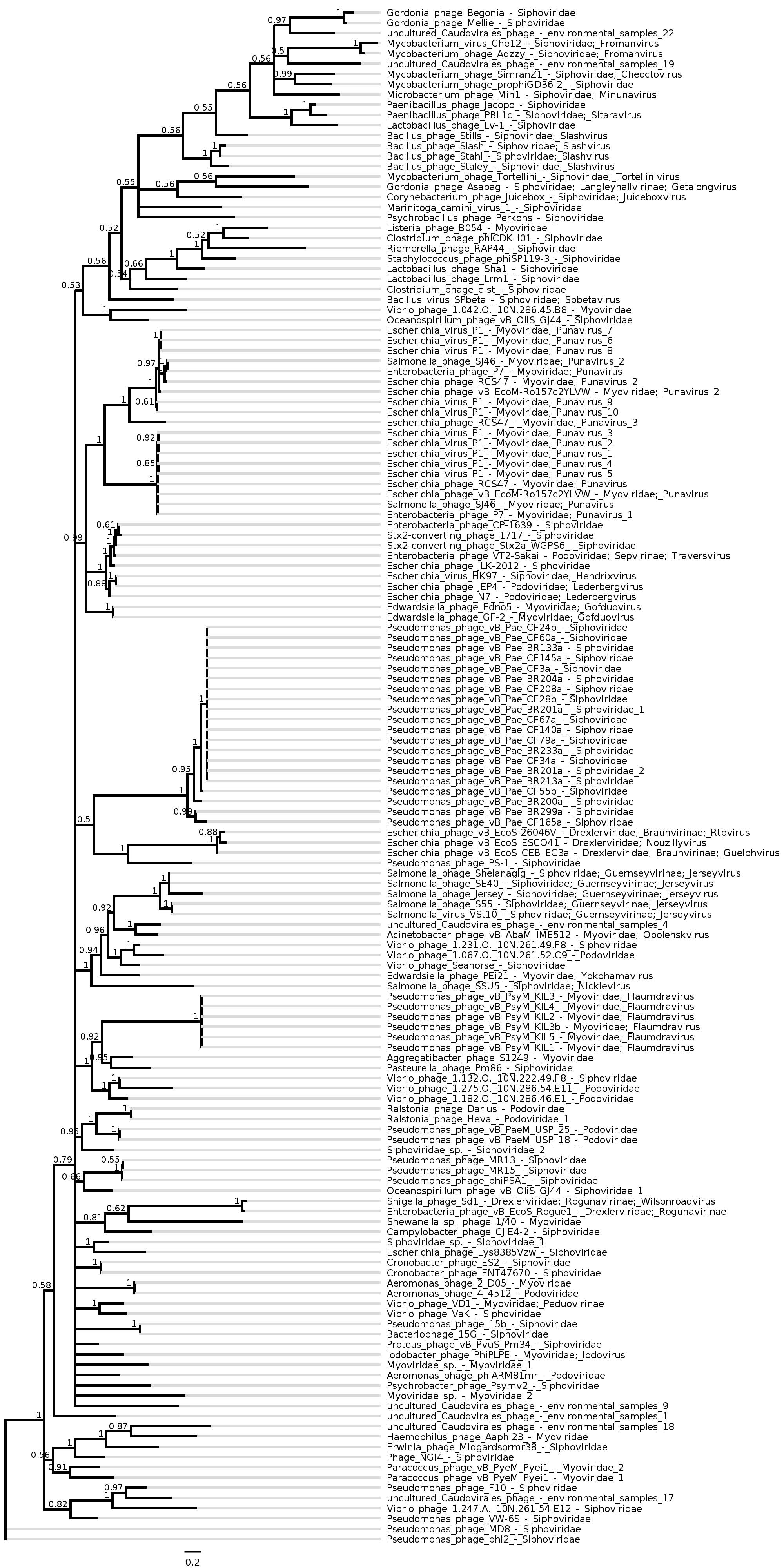

Supplement: Supplementary file 1 [file ijms-22-10350-s001.zip › SF11_gp55_antirepressor protein CII_0893.jpg]

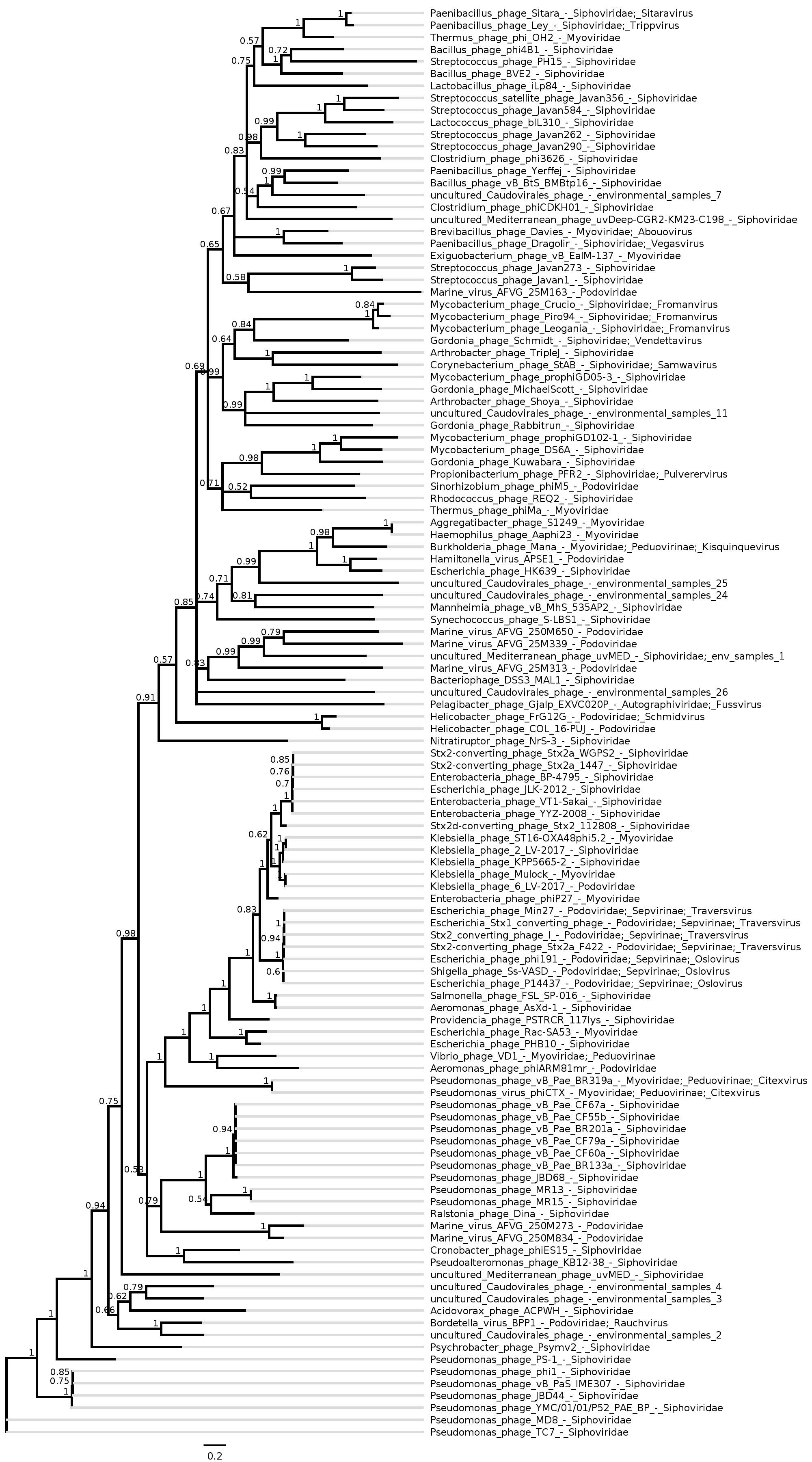

Supplement: Supplementary file 1 [file ijms-22-10350-s001.zip › SF12_gp33_integrase_0269.jpg]

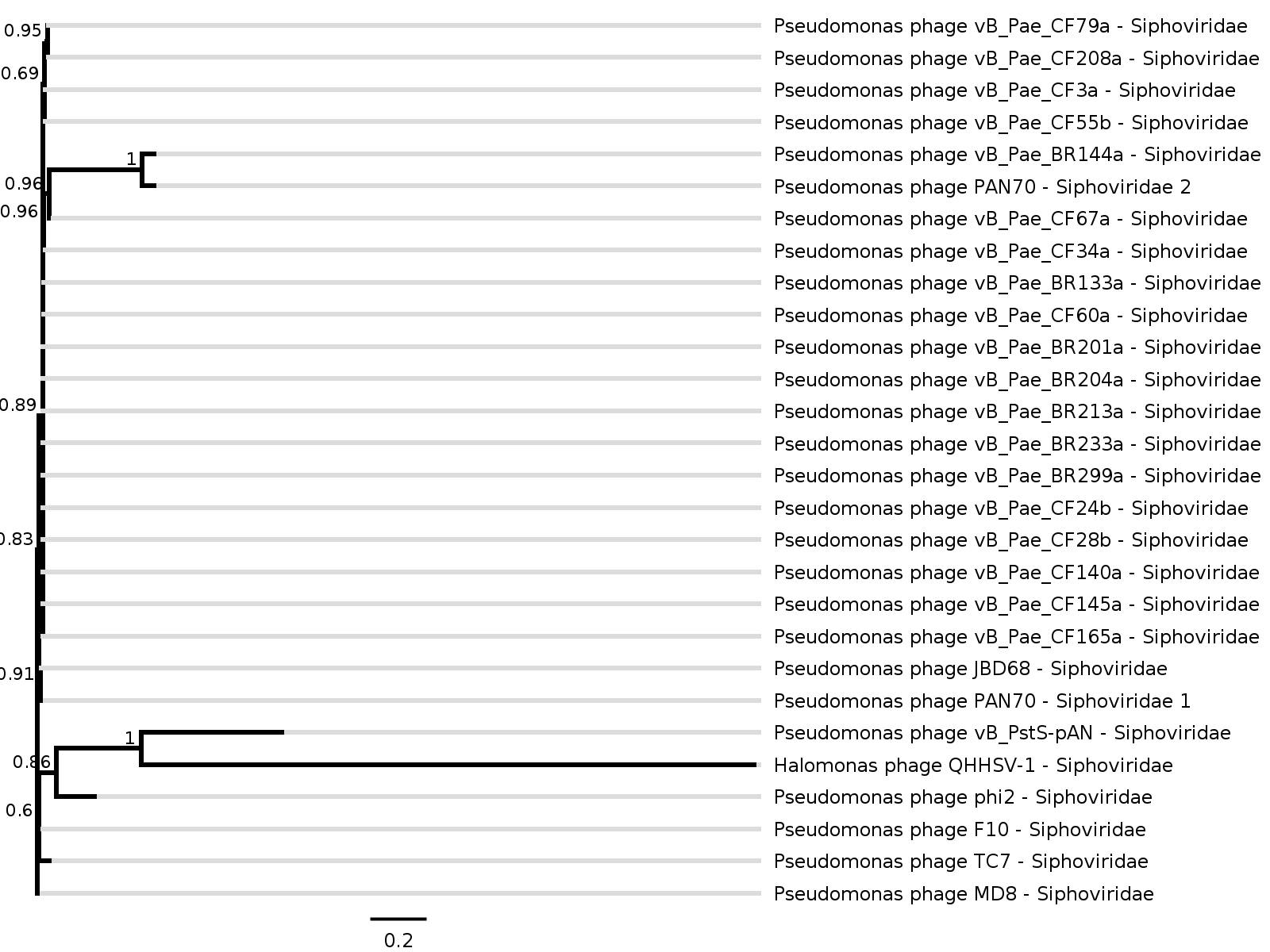

Supplement: Supplementary file 1 [file ijms-22-10350-s001.zip › SF13_gp18_tail tip protein_0107.jpg]

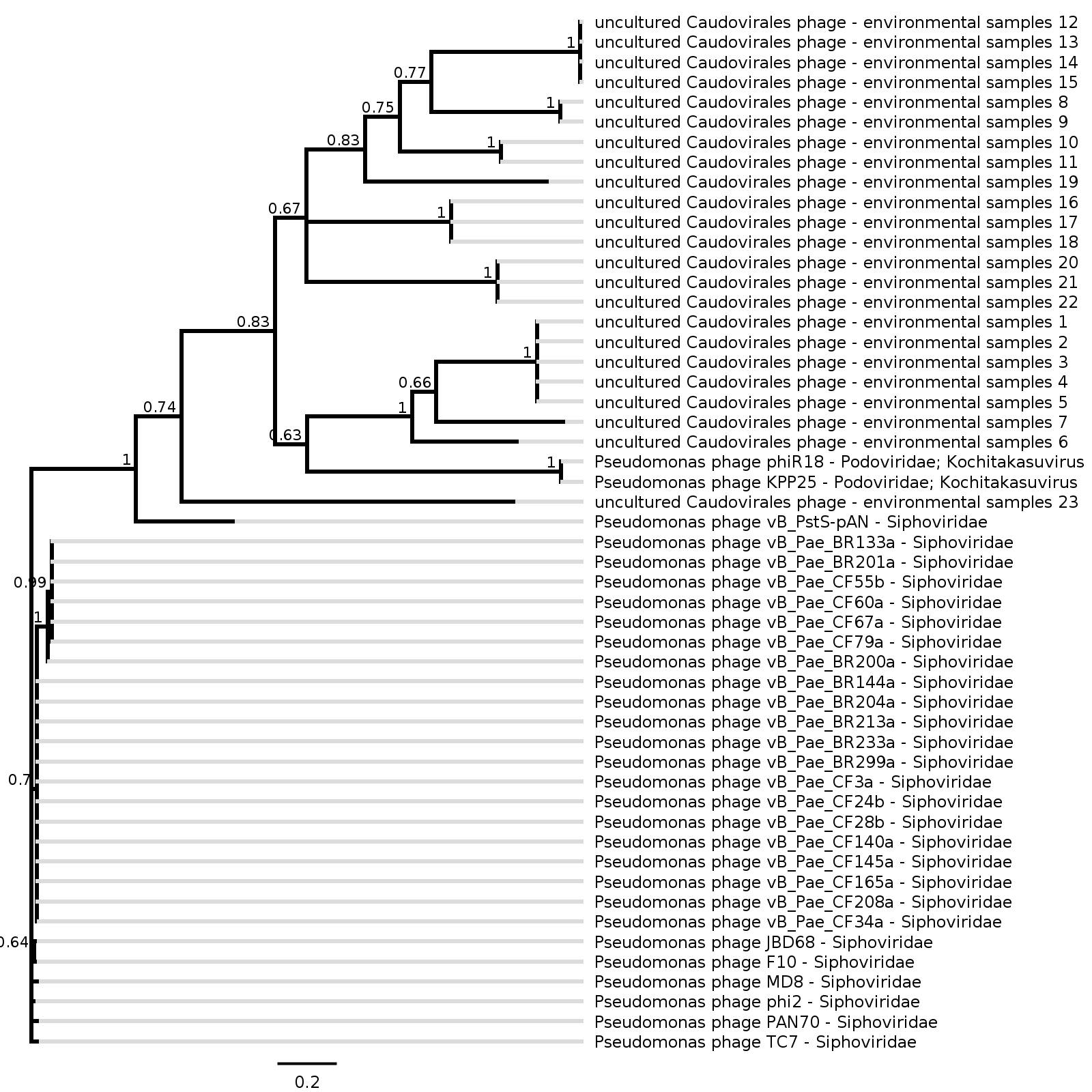

Supplement: Supplementary file 1 [file ijms-22-10350-s001.zip › SF14_gp23_tail fiber protein_0052.jpg]

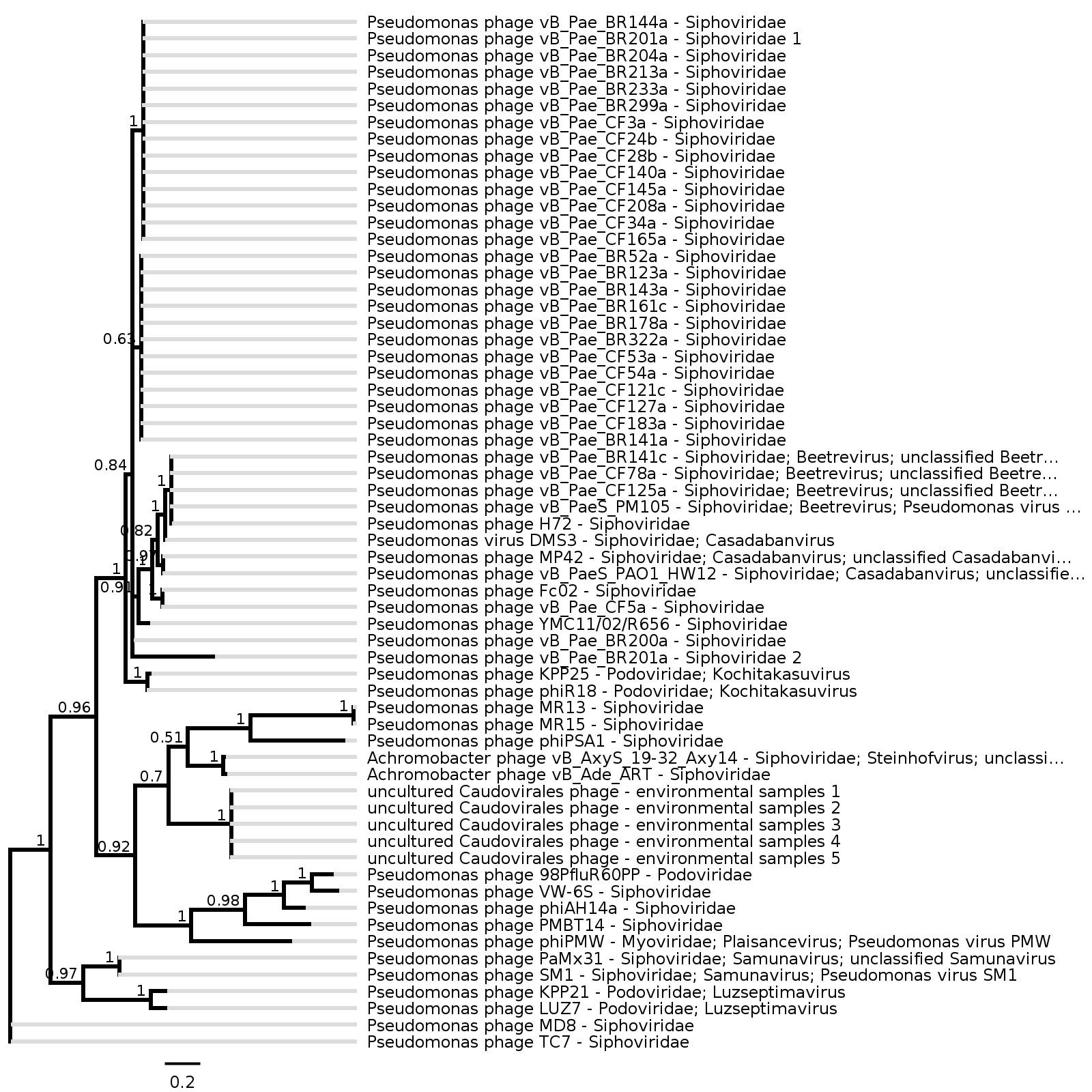

Supplement: Supplementary file 1 [file ijms-22-10350-s001.zip › SF15_gp28_tail fiber protein_0038.jpg]

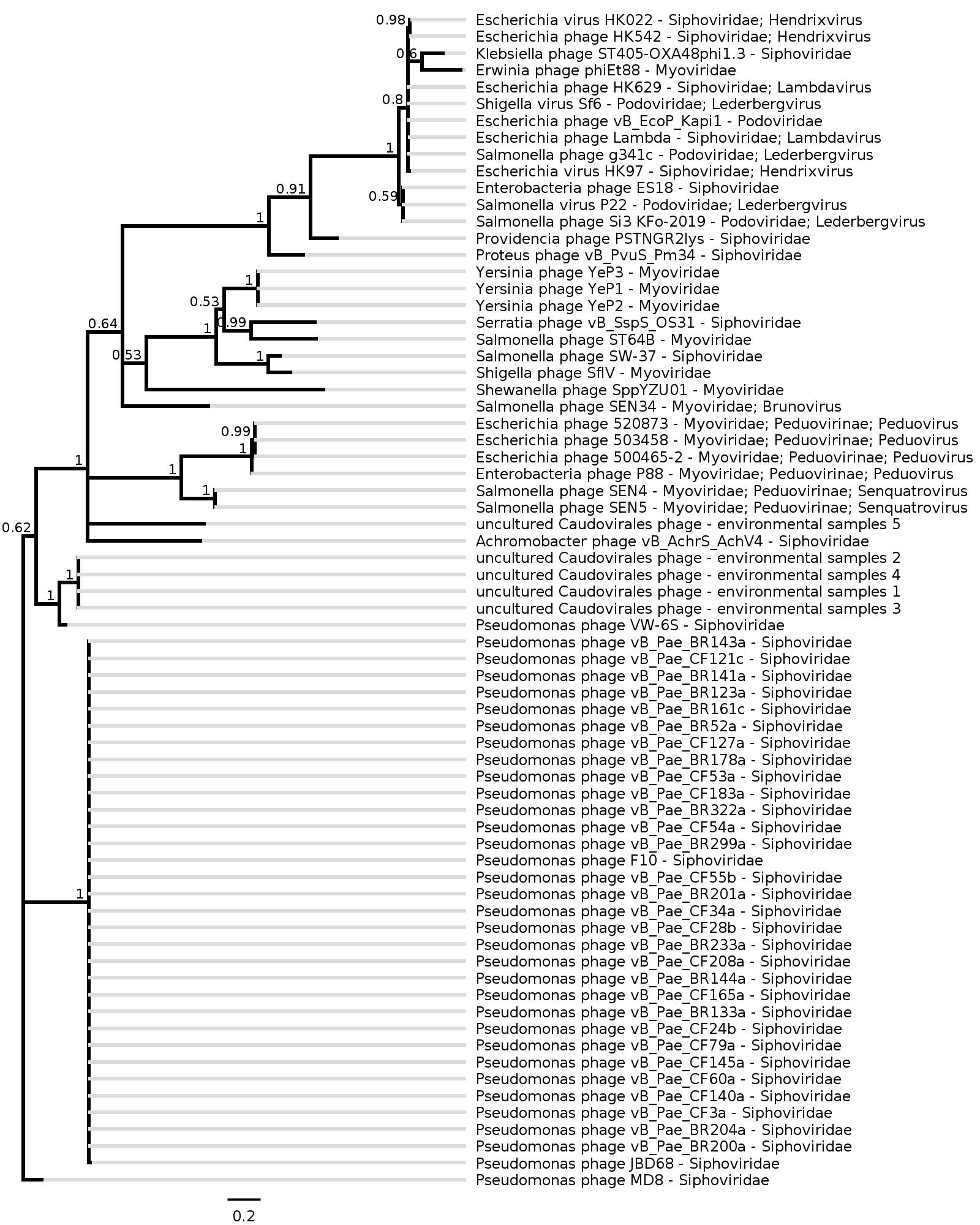

Supplement: Supplementary file 1 [file ijms-22-10350-s001.zip › SF16_gp62_holin_0088.jpg]

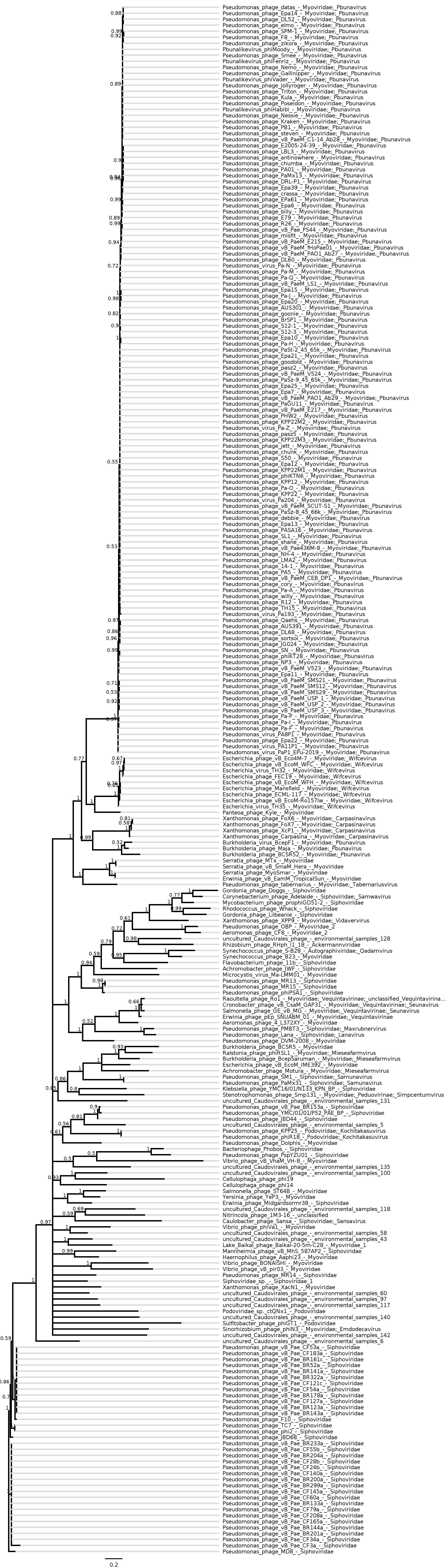

Supplement: Supplementary file 1 [file ijms-22-10350-s001.zip › SF17_gp63_endolysin_0936.jpg]

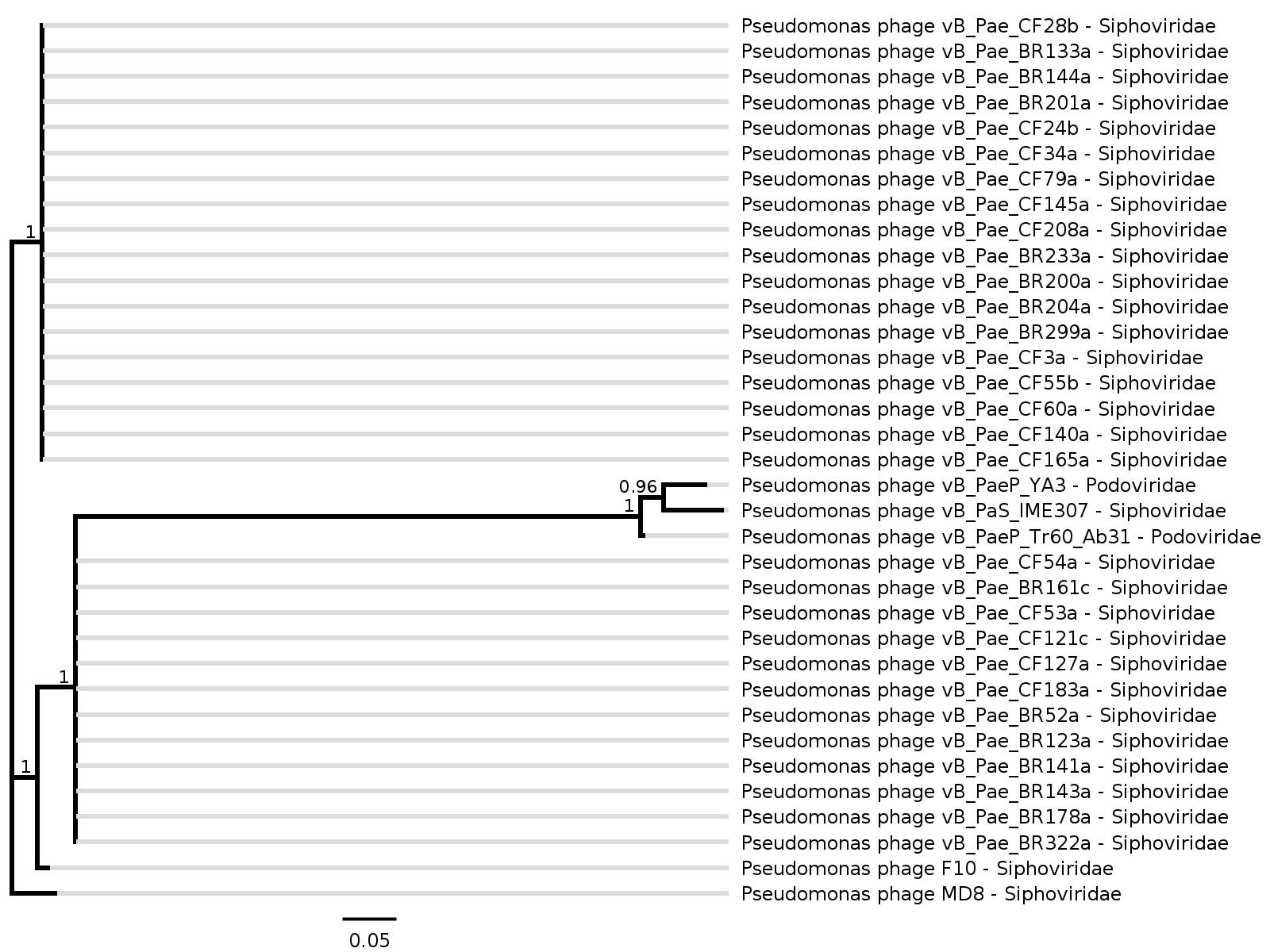

Supplement: Supplementary file 1 [file ijms-22-10350-s001.zip › SF18_gp65_i-spanin_0019.jpg]

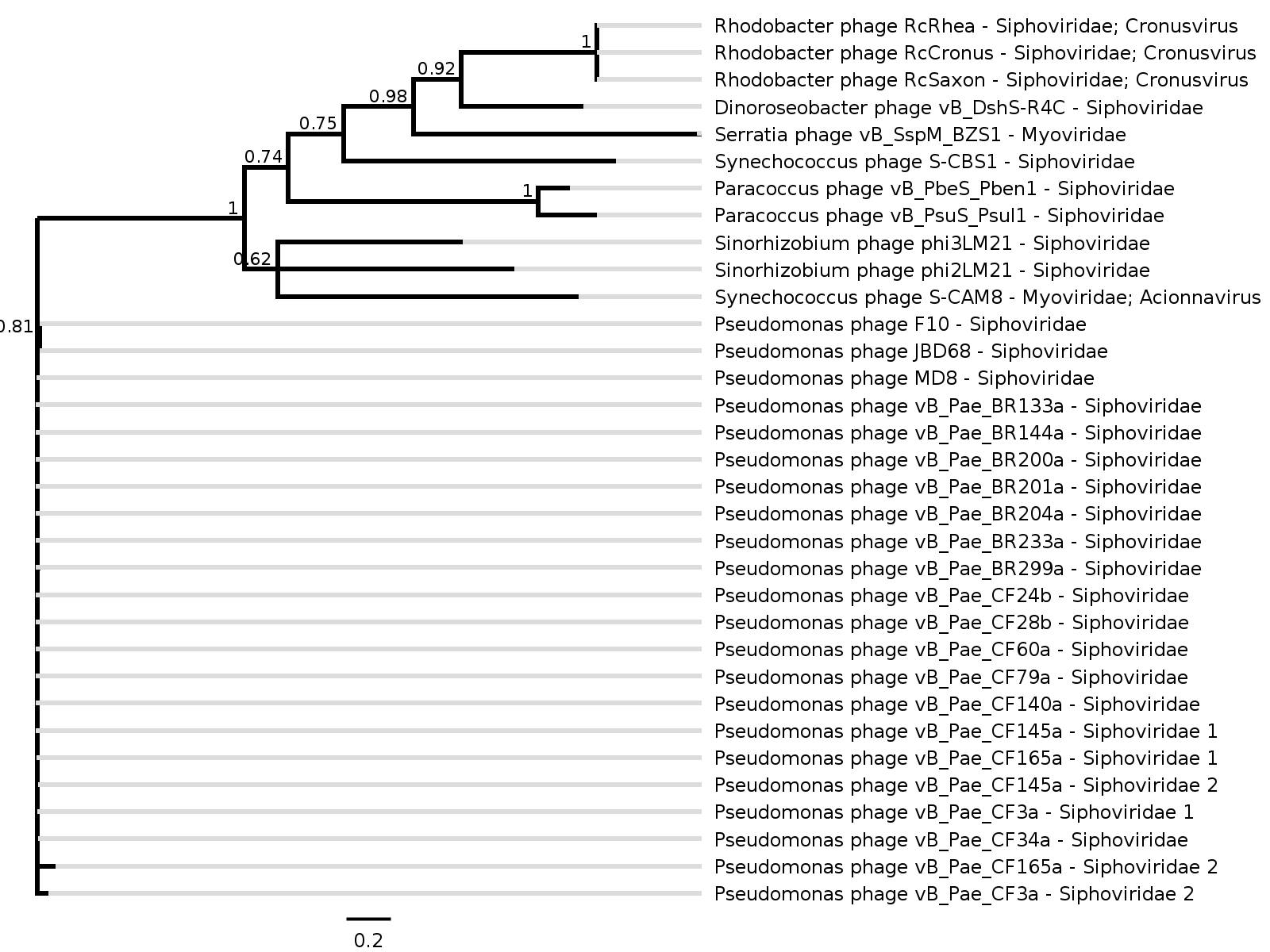

Supplement: Supplementary file 1 [file ijms-22-10350-s001.zip › SF19_gp66_o-spanin_0084.jpg]

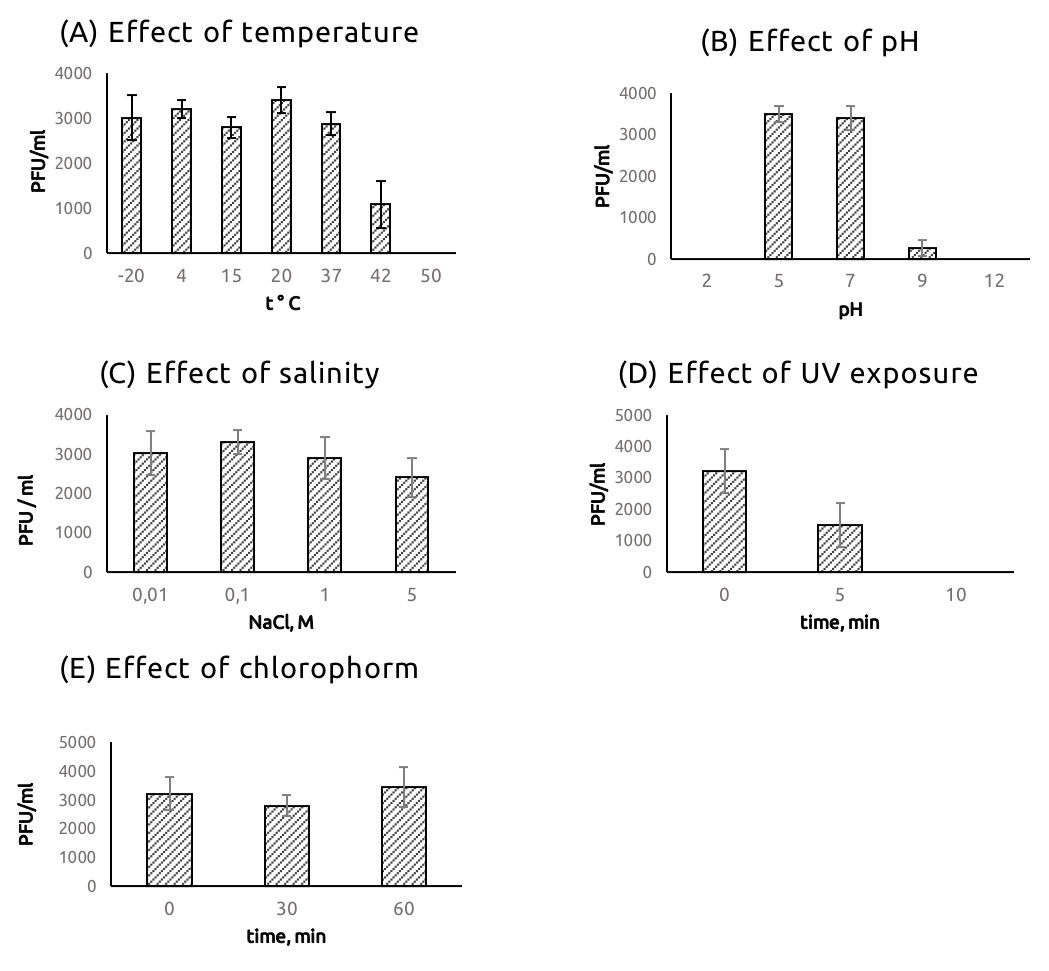

Supplement: Supplementary file 1 [file ijms-22-10350-s001.zip › SF2_cropped.jpg]

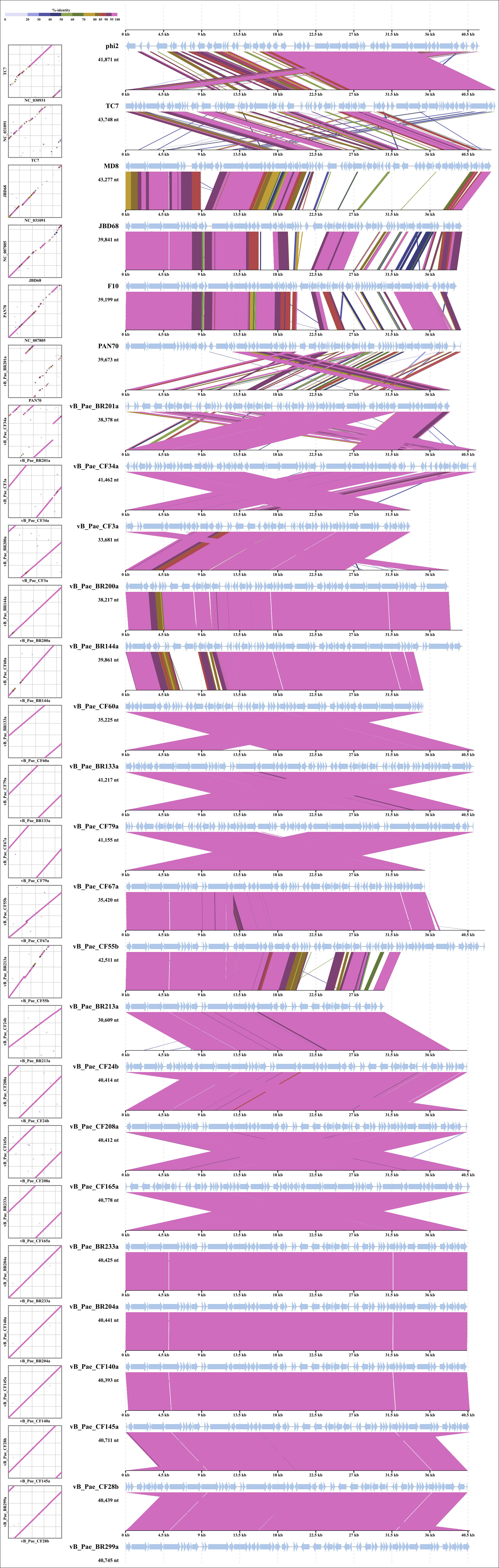

Supplement: Supplementary file 1 [file ijms-22-10350-s001.zip › SF20_vip-compar.jpg]

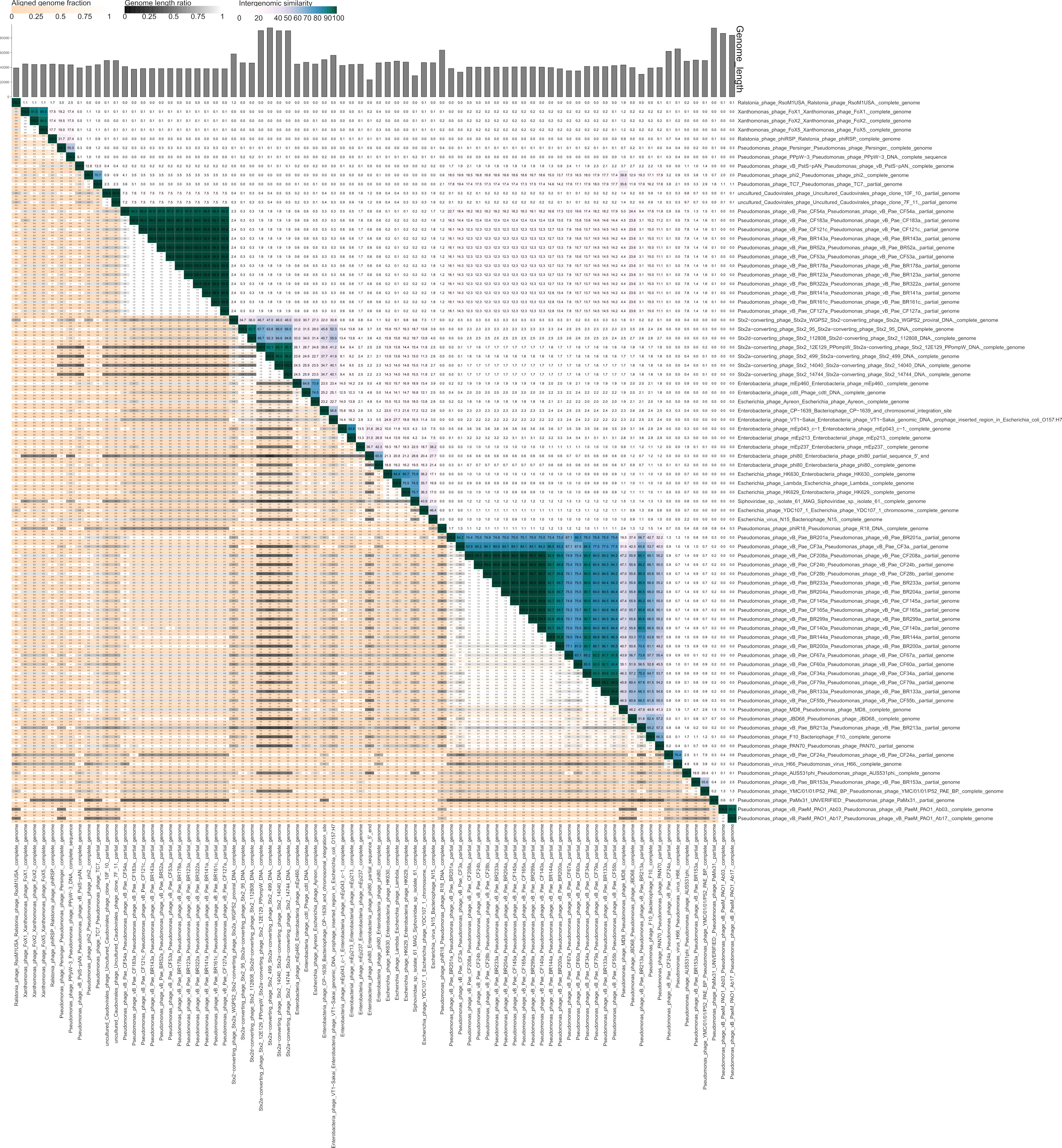

Supplement: Supplementary file 1 [file ijms-22-10350-s001.zip › SF3_VIRIDIC-80.jpg]

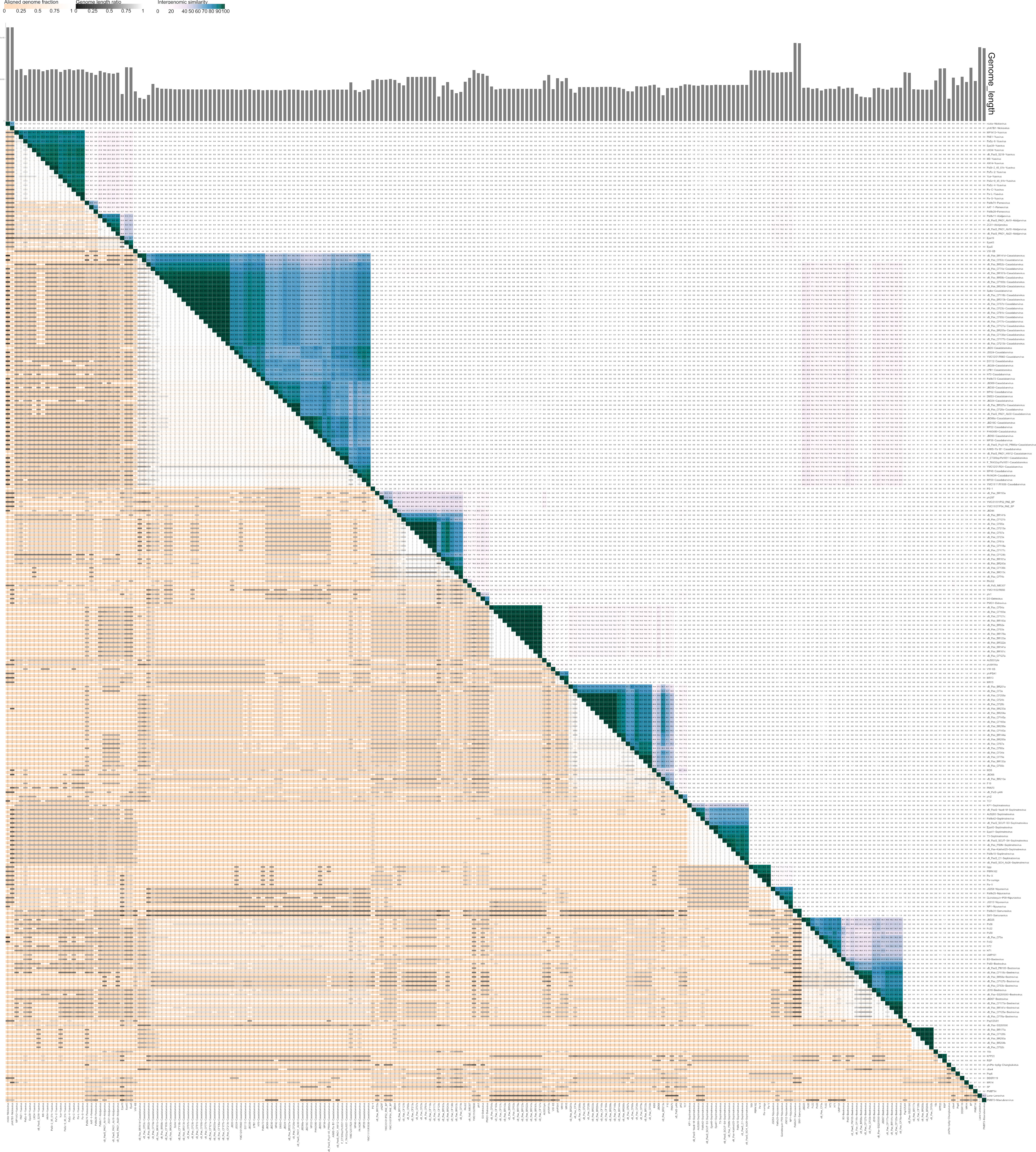

Supplement: Supplementary file 1 [file ijms-22-10350-s001.zip › SF4_VIRIDIC-224.jpg]

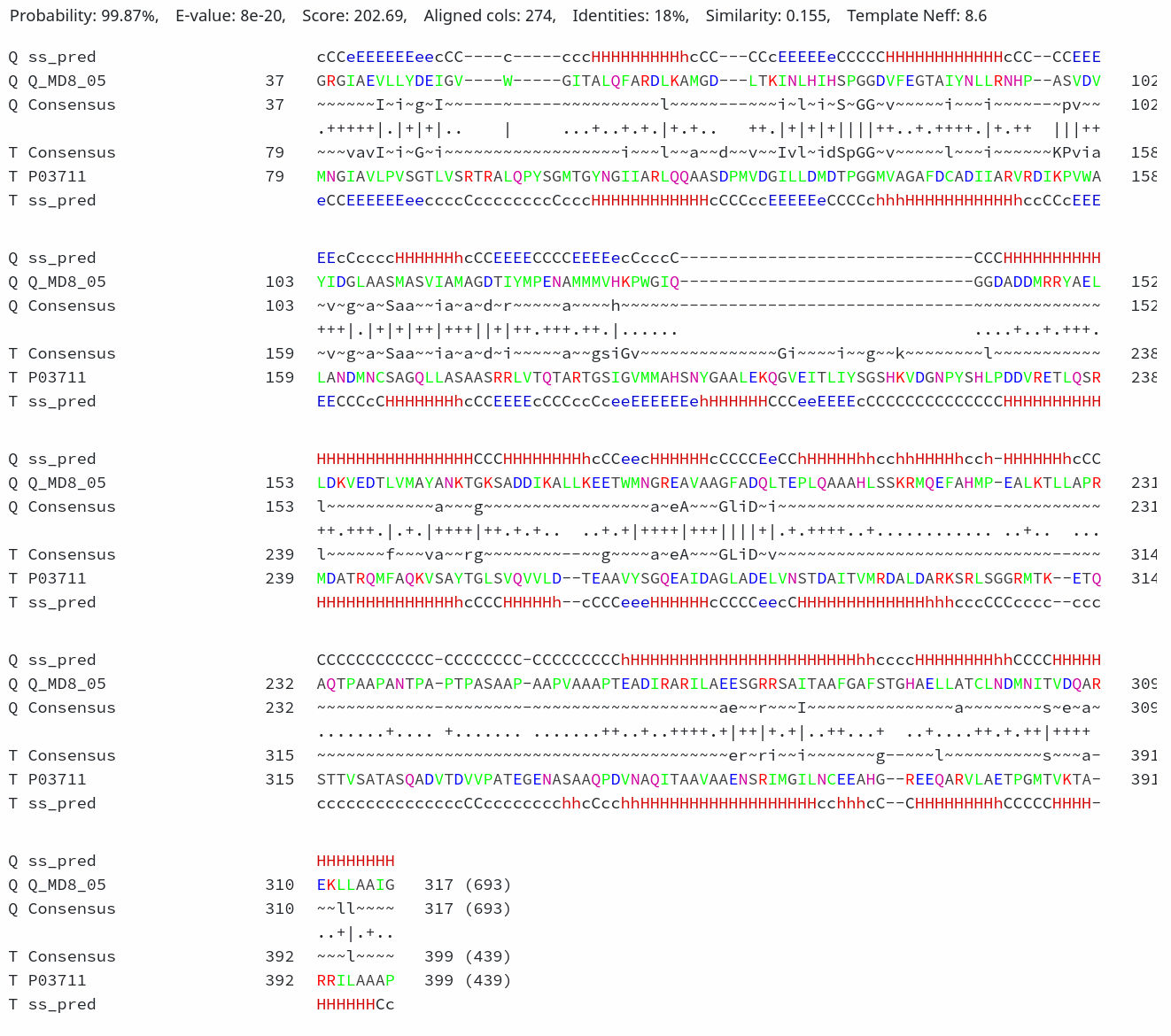

Supplement: Supplementary file 1 [file ijms-22-10350-s001.zip › SF5_HHpred_N-end_gp5.jpg]

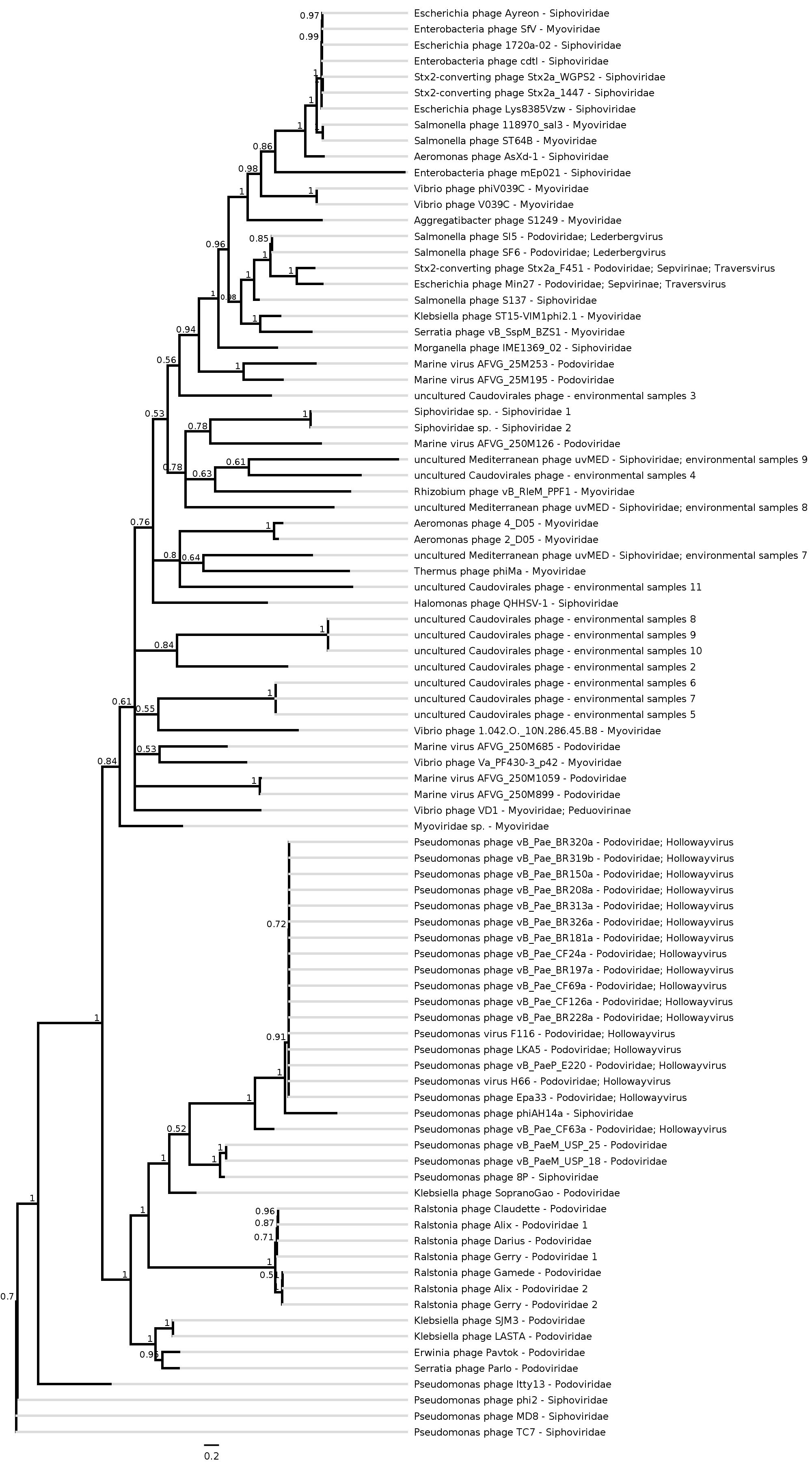

Supplement: Supplementary file 1 [file ijms-22-10350-s001.zip › SF6_gp56_replication protein O_0130.jpg]

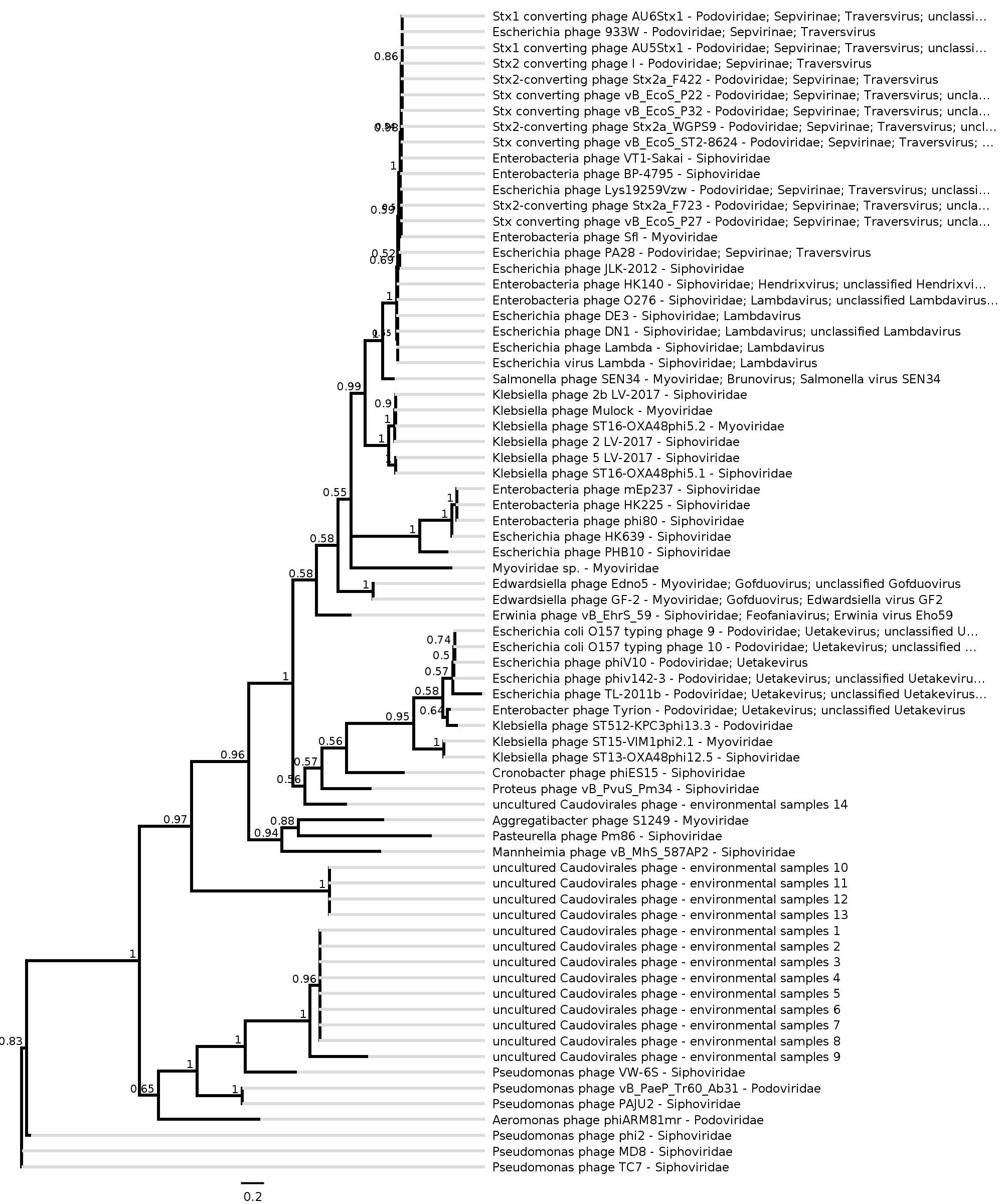

Supplement: Supplementary file 1 [file ijms-22-10350-s001.zip › SF7_gp57_replication protein P_0207.jpg]

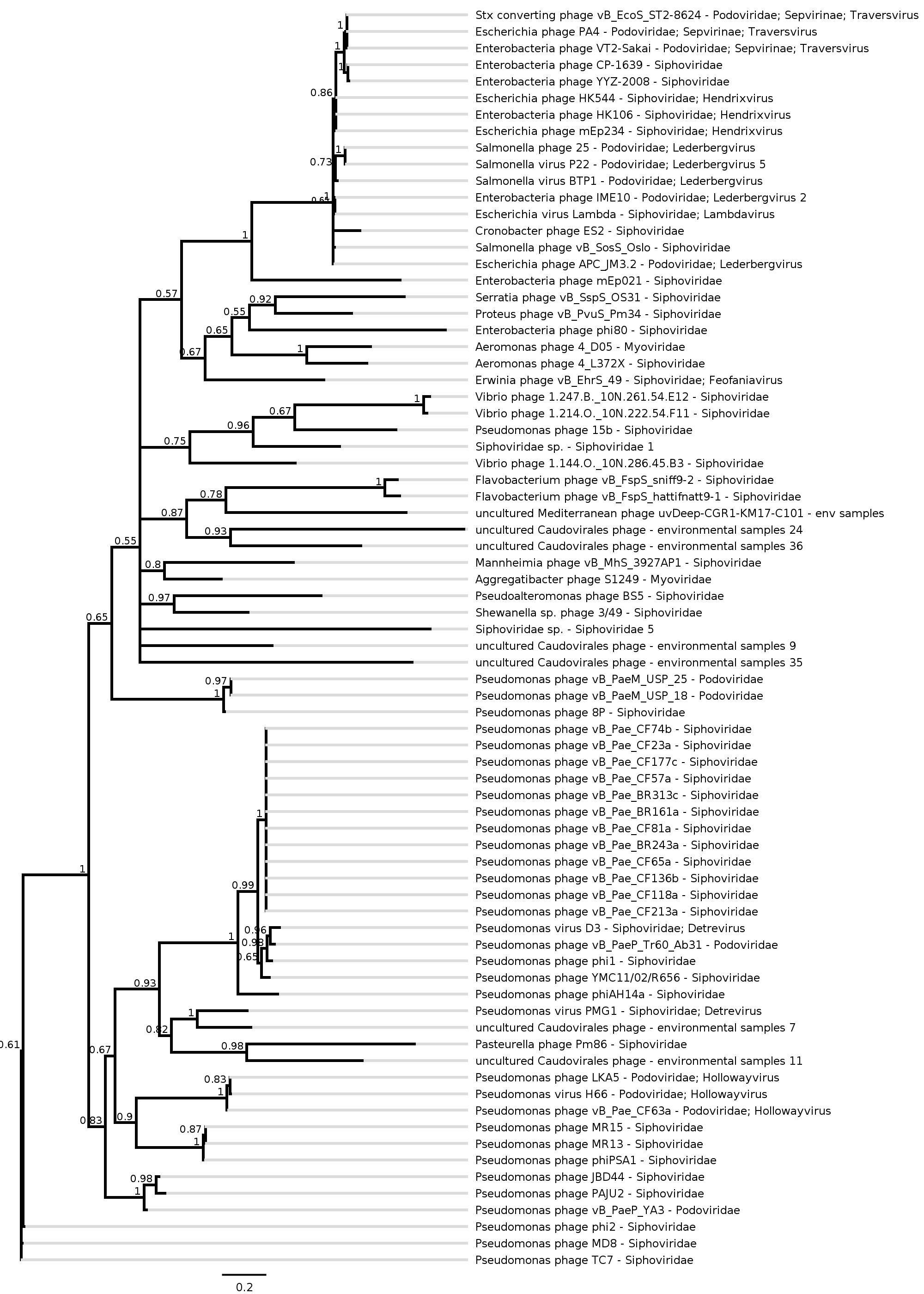

Supplement: Supplementary file 1 [file ijms-22-10350-s001.zip › SF8_gp59_NinG recombination protein_0526.jpg]

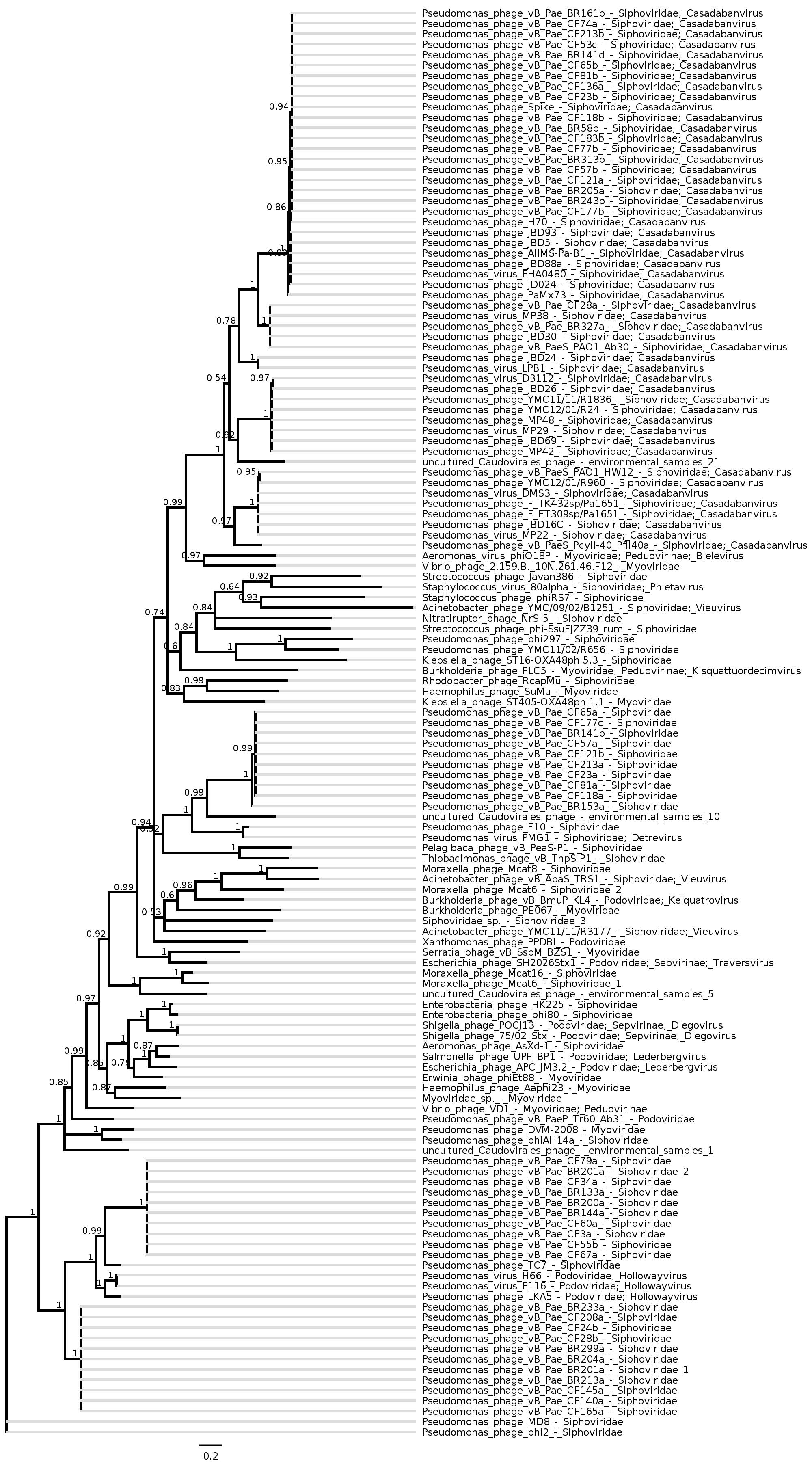

Supplement: Supplementary file 1 [file ijms-22-10350-s001.zip › SF9_gp53_repressor protein cI_0107.jpg]
